# Genome instability-related LINC02577, LINC01133 and AC107464.2 are lncRNA prognostic markers correlated with immune microenvironment in pancreatic adenocarcinoma

**DOI:** 10.1186/s12885-023-10831-4

**Published:** 2023-05-12

**Authors:** Yinjiang Zhang, Yao Wang, Xu He, Rongfei Yao, Lu Fan, Linyi Zhao, Binan Lu, Zongran Pang

**Affiliations:** 1 School of Pharmacy, Minzu University of China, No. 27, Zhongguancunnan Street, Haidian District, Beijing, 100081 People’s Republic of China; 2 Key Laboratory of Ethnomedicine, Ministry of Education, Minzu University of China), Beijing, People’s Republic of China; 3 Department of Radiation Oncology, Chinese PLA General Hospital, Beijing, 100853 China

**Keywords:** Pancreatic adenocarcinoma, Genome instability, Immune checkpoint, Immunotherapy, LncRNA

## Abstract

**Background:**

Pancreatic adenocarcinoma (PAAD) is a leading cause of malignancy-related deaths worldwide, and the efficacy of immunotherapy on PAAD is limited. Studies report that long non-coding RNAs (lncRNAs) play an important role in modulating genomic instability and immunotherapy. However, the identification of genome instability-related lncRNAs and their clinical significance has not been investigated in PAAD.

**Methods:**

The current study developed a computational framework for mutation hypothesis based on lncRNA expression profile and somatic mutation spectrum in pancreatic adenocarcinoma genome. We explored the potential of GInLncRNAs(genome instability-related lncRNAs) through co-expression analysis and function enrichment analysis. We further analyzed GInLncRNAs by Cox regression and used the results to construct a prognostic lncRNA signature. Finally, we analyzed the relationship between GILncSig (genomic instability derived 3-lncRNA signature) and immunotherapy.

**Results:**

A GILncSig was developed using bioinformatics analyses. It could divide patients into high-risk and low-risk groups, and there was a significant difference in OS between the two groups. In addition, GILncSig was associated with genome mutation rate in pancreatic adenocarcinoma, indicating its potential value as a marker for genomic instability. The GILncSig accurately grouped wild type patients of KRAS into two risk groups. The prognosis of the low-risk group was significantly improved. GILncSig was significantly correlated with the level of immune cell infiltration and immune checkpoint.

**Conclusions:**

In summary, the current study provides a basis for further studies on the role of lncRNA in genomic instability and immunotherapy. The study provides a novel method for identification of cancer biomarkers related to genomic instability and immunotherapy.

**Supplementary Information:**

The online version contains supplementary material available at 10.1186/s12885-023-10831-4.

## Introduction

Pancreatic adenocarcinoma (PAAD) is a malignancy associated with high mortality rates, with a 5-year survival rate of 8% [1]. Although advances in surgery, chemotherapy and immunotherapy have improved treatment of PAAD, the 5-year survival rate is still low [2, 3]. Diagnosis of PAAD is challenging owing to specific clinical manifestations in the early stage. Approximately 80% of PAAD patients present with advanced stage at the time of diagnosis thus limiting the efficacy of treatment [4]. Therefore, establishing specific biomarkers is important for diagnosis and prognosis evaluation of pancreatic adenocarcinoma.

Genomic instability is a marker of various cancer types [5]. In addition, genomic instability is an important prognostic factor. High genomic instability is correlated with tumor progression and survival [6, 7]. Although the molecular basis of genomic instability has not been fully elucidated, transcriptional abnormalities and post-transcriptional regulation are correlated with genomic instability [8]. This indicates that molecular markers have the potential to be a quantitative measure of genome instability. Habermann et al. [9] developed a genomic instability signature of 12 genes by exploring the gene expression profile of 48 breast cancer specimens.

DNA replication, DNA damage repair and chromosome segregation are the main sources of genomic instability and are the leading causes of tumorigenesis. Studies report that detection of germ cell mutations, somatic mutations and fusion, and microsatellite instability (MSI)/mismatch repair defects in pancreatic adenocarcinoma play a key role in optimizing treatment options and conducting clinical trials on targeted drugs [10]. Genome-wide studies on PAAD have reported several genes that may aggravate genomic instability, such as BRCA 1, BRCA 2, palb 2, ATM, ATR, arid1a, Chek 1 / 2, RPA 1 and MMR genes [11, 12]. Moreover, mutational phenomena, such as chromothripsis and polyploidization, are associated with unstable tumors and aggressive tumor behavior. PAAD exhibits a high frequency of chromothripsis and polyploidization, implying that they play a role in development and progression of PAAD. Therefore, identification of genomic instability related molecules in PAAD can provide new drug targets and markers, and provide information on diagnosis and prognosis of PAAD [10]. This is a promising area for pancreatic adenocarcinoma research, which can lead to developing personalized therapies and improving the treatment of pancreatic adenocarcinoma. In addition, tumor characteristics based on genomic instability, including invasion and metastasis, metabolic recombination and immune escape, make tumor treatment challenging [13]. PD-1/PD-L1 checkpoint inhibition has been reported in various malignant tumors [14, 15]. However, studies report that patients with PAAD show poor response to PD-1 antibody [16]. Pu et al. [17] explored the relationship between genomic instability and anti-tumor immunity. The findings showed that genomic stress is a promising strategy to restore anti-tumor immunity, thus providing new opportunities for cancer treatment. These studies indicate that the effect of immune therapy on pancreatic adenocarcinoma can be improved by modulating genomic instability.

Long non-coding RNAs (lncRNAs) are broadly defined as transcripts with more than 200 nt and have low protein-coding potential [18]. LncRNAs play an important role in different biological processes [19, 20]. Although studies report lncRNAs play a key role in regulating genomic instability and tumor immune microenvironment, the clinical significance of genomic instability related lncRNAs in PAAD and their relationship with tumor immune microenvironment have not been explored.

A 3-lncRNA signature associated with genomic instability at the genomic and transcriptional levels was developed in the current study. Further, the study explored the prognostic significance in PAAD patients and their relationship with tumor immune microenvironment. The findings of the current study provide a theoretical basis for improving pancreatic adenocarcinoma immunotherapy through the regulation of genomic instability of lncRNAs.

## Materials and methods

### Data retrieval

TCGA-PAAD cohort was obtained from the TCGA database (https://portal.gdc.cancer.gov/projects/TCGA-PAAD). RNAseq expression data was retrieved in FPKM format, whereas corresponding clinical data was obtained in BCR XML format. Further, somatic cell mutation information was obtained. RNA-seq expression data was annotated using Gencode v22 tool. Moreover, expression matrixes of lncRNA and mRNA were extracted. Download the GSE78220 data set [21] of anti-PD-1 therapy in melanoma and the corresponding clinical information from the GEO database. Data were analyzed with the R and R Bioconductor packages.

### LncRNAs differential analysis and sample typing

The top 25% of patients (*n* = 43) were defined as genomic unstable (GU) and the last 25% of patients (*n* = 40) were defined as genomic stable (GS) based on the cumulative number of somatic mutations. lncRNA expression of 43 patients in the GU sample group and 40 patients in the GS sample group were compared. LncRNA expression matrix was constructed using limma [22] package in R (3.6.0), and differences in expression level were analyzed by Wilcox test. P values were corrected by FDR method (FDR < 0.05, logFC > 1). A heat map of the top 20 most significantly upregulated lncRNAs and the most significantly downregulated lncRNAs was generated using Pheatmap package [23]. A total of 178 TCGA samples were classified by hclust function in R based on differentially expressed lncRNAs. Correlation between sample typing and mutation and UBQLN4 gene expression was analyzed and visualized using ggboxplot function.

### Co-expression network analysis of lncRNAs

Limma in R was used to analyze co-expression of differentially expressed lncRNAs and mRNAs. The top 10 mRNAs with the highest correlation with lncRNAs were identified. Co-expression network of lncRNAs and mRNAs was visualized using igraph [24] package.

### GO and KEGG enrichment analysis

Org.Hs.eg.db [25] package in R was used to convert the gene symbol of lncRNA co-expressed genes into gene ID. Clusterprofiler [26] and enrichplot packages were used for enrichment analysis, and ggplot2 [27] was used for visualization. Terms with a p value and q value less than 0.05 were considered to be significantly enriched.

### LncRNAs prognostic model construction

Correlation of differentially expressed lncRNA with the corresponding TCGA clinical survival time was analyzed using limma package. Patients were randomly divided into training set (87 patients) and testing set (84 patients). Univariate and multivariate Cox proportional hazard regression analyses were used to identify the prognostic lncRNAs in the training set and the findings were used to construct a prognostic risk model. The testing set was used to independently validate the prognostic risk model. Risk scores for the training set, testing set and TCGA set predicted by the model were determined. Chi-square test was used to explore differences in clinical traits between training set and testing set, and to verify whether there was clinical bias in sample grouping.

### Survival and ROC analysis

Patients were classified into high-risk group or low-risk group based on the median score of patients as the risk cutoff. Survival analysis was performed on patients in the training set, testing set and TCGA set using the survival package [28] and survminer package in R [29]. The ggsurvplot function was used to visualize the survival curve. One-year ROC curves were generated using timeROC [30] package.

### Risk curve analysis

Limma package in R was used to analyze the relationship between patient risk and gene expression and genomic instability in the training set, testing set and TCGA set. Pheatmap was used to generate patient risk heat maps, and the plot function was used to plot mutation graphs and UBQLN4 expression graphs. The ggpubr package was used to explore the correlation between mutations and UBQLN4 expression in patients in the high and low risk groups. The boxplot function in R was used for visualization.

### Independent prognostic analysis

Univariate analysis and multivariate independent prognostic analysis of the training set, testing set and TCGA set were performed. The results were then used to construct a prognosis model using the survival package in R.

### Clinical grouping model validation

Survival and survminer packages in R were used to verify whether the prognosis model applied to patients in different clinical groups. Plyr [31] package was used to analyze the relationship between high and low risk groups, and KRAS gene mutation and the chi-square test was performed to explore differences between the two groups.

Survival and survminer packages were used to analyze KRAS gene mutations in different genotypes. The prognostic model that was constructed was compared with models constructed by Shilncsig [32], Weilncsig [33] and Wulncsig [34].

### Analysis of the relationship between GILncSig and TME

The relative abundance of PAAD TME (tumor immune microenvironment) cells was quantified using the ssGSEA algorithm (single-sample gene-set enrichment analysis). In the study of Charoentong [35], the gene set for marking each type of TME infiltration immune cell was obtained. Each sample was analyzed based on its enrichment scores calculated by the ssGSEA analysis. PAAD patients' immune and ESTIMATE scores were calculated using the 'estimate' R package [36].

### GILncSig-based treatment approach for PAAD

The correlation of GILncSig scores with immune checkpoints, histocompatibility complexes and immunostimulators was analyzed in the TCGA-PAAD by the ggpubr package. Based on GSE78220 normalized data, we calculated the GILncSig scores and analyzed its impact on prognosis and PD-1 inhibitor efficacy. Using the pRRophetic R package [37], semi-inhibitory concentrations (IC50s) for every PAAD patient in the TCGA cohort were predicted.

### Correlation analysis of 3-lncRNA expression level and clinicopathological features

Toil process [38] transformed TCGA and GTEx datasets into TPM RNAseq data format and were retrieved from UCSC Xena webserver (https://xenabrowser.net/datapages/). Pancreatic adenocarcinoma data were retrieved from TCGA and matched normal tissue data were retrieved from the GTEx portal. Correlation between lncRNA prognostic models (LINC02577, LINC01133 and AC107464.2) and clinicopathological characteristics of patients with pancreatic adenocarcinoma were analyzed. In addition, differential expression levels between tumor and normal tissues were analyzed. Wilcoxon rank sum test was used for comparing differences between two groups, whereas Kruskal–Wallis test was used to compare more than three groups. pROC package [39] was used to construct the diagnostic ROC curve.

According to Moffitt et al. [40] and Collisson et al. [41], TCGA-PAAD samples were divided into two subtypes and three subtypes, respectively. The differential expression of the three lncRNAs in Moffitt and Colisson's classification was analyzed. GSE133684 [42] is a blood extracellular vesicle lncRNA sequencing data set consisting of 284 PDAC patients and 117 healthy controls. Standardize the RNA-seq data of GSE133684 with log2(TPM + 1). We analyzed the expression levels of three lncRNAs in blood samples.

### 3-lncRNA GSEA analysis

Correlations between expression of the 3 lncRNAs and all genes were explored using R. GSEA analysis was then performed using the clusterProfiler [26] package in R. |ES|> 1, p.adjust < 0.05, and FDR < 0 0.25 represented statistical significance.

### Analysis of 3-lncRNA and immune cell infiltration

GSVA package [43] was used to perform Spearman’s correlation between 3-lncRNA and 24 types of immune cells [44] using ssGSEA immune cell algorithm. SIGLEC15, IDO1, CD274, HAVCR2, PDCD1, CTLA4, LAG3 and PDCD1LG2 were used as immune checkpoint-related transcripts. Expression values of these eight genes were determined, and Spearman’s correlation analysis was performed between 3-lncRNA and immune checkpoint-related genes. *P* < 0.05 was considered statistically significant. Subcellular localization of lncRNAs was predicted using lncLocator tool [45] (http://www.csbio.sjtu.edu.cn/bioinf/lncLocator/).

## Results

### Study design

**Fig. 1:**
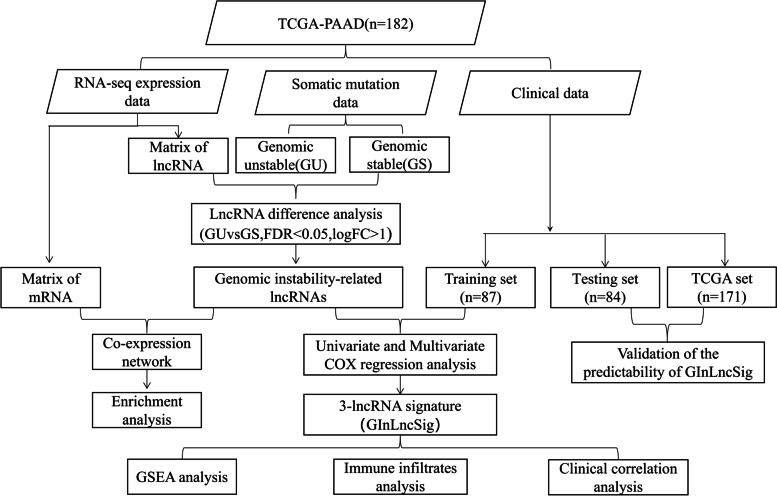
Study design. PAAD: Pancreatic adenocarcinoma

The study design is presented in Fig. 1. GInLncRNAs were identified in combination with somatic mutations and transcriptome data. We explored the potential of GInLncRNAs through co-expression analysis and function enrichment analysis. The patient cohort was then randomized into three data sets for subsequent analysis, including training, testing, and TCGA sets. We further analyzed GInLncRNAs by Cox regression and used the results to construct a prognostic lncRNA signature. Finally, we analyzed the relationship between GILncSig (genomic instability derived 3-lncRNA signature) and immunotherapy.

### Identification of genome instability associated lncRNAs in PAAD patients

**Fig. 2:**
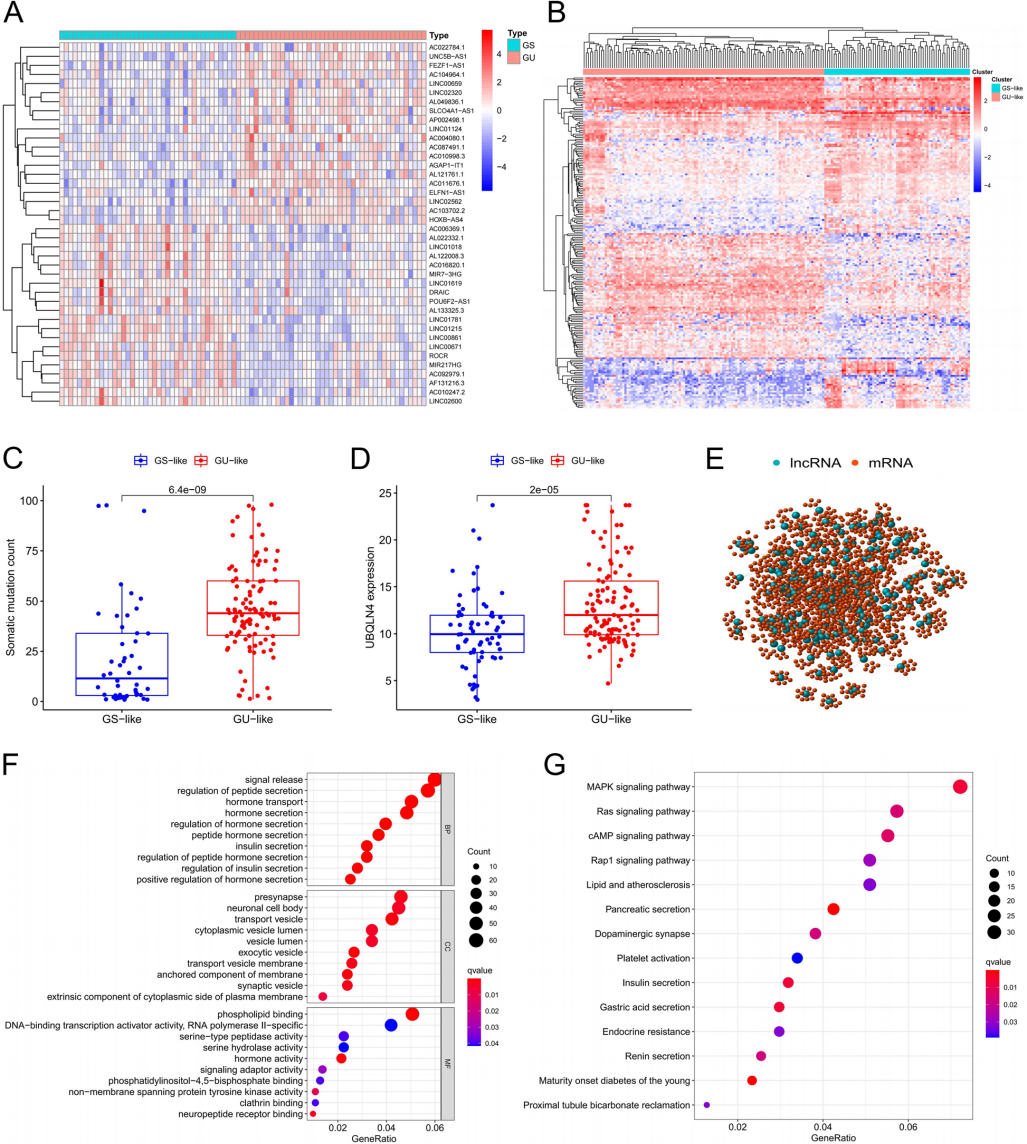
Identification and functional annotation of lncRNAs associated with genomic instability in PAAD patients. **A** Heatmap of top 20 genomic instability-related lncRNAs with the most significant upregulated and downregulated lncRNAs. **B** Based on 189 lncRNAs associated with genomic instability, unsupervised clustering was performed in 178 PAAD patients. The blue cluster on the right represents the GS-like group, and the red cluster on the left represents the GU-like group. **C** Somatic cumulative mutations in the GU-like group are significantly higher compared with those in the GS-like group. **D** The expression level of UBQLN4 in GU-like group was significantly higher than that in GS-like group. **E** Based on Pearson correlation coefficient, we constructed a network of genomic instability-related lncRNAs and their co-expressed genes. Blue circle represents lncRNA, and red circle represents mRNA. **F-G** The GO and KEGG functions of lncRNA co-expressed mRNAs were analyzed

To explore genome instability-related lncRNAs, a total of 189 lncRNAs (FDR < 0.05, logFC > 1) were isolated from pancreatic adenocarcinoma samples. Patients were grouped as genomic unstable (GU) group and genomic stable (GS) group. Analysis showed that the expression levels of 90 lncRNAs were up-regulated and 99 down-regulated in the GU group (Table S1). A heat map was constructed for the top 20 most significantly upregulated lncRNAs and top 20 downregulated 20 lncRNAs (Fig. 2A). Unsupervised hierarchical cluster analysis was performed on 178 samples from the TCGA set based on 189 differentially expressed lncRNAs. The samples were divided into two groups including a group with higher cumulative somatic mutations (GU-like) and a group with lower cumulative somatic mutations (GS-like) (Fig. 2B). Analysis showed that the median value of cumulative somatic mutations in the GU-like group was significantly higher than that in the GS-like group (Fig. 2C, *P* < 0.001). In addition, the expression level of UBQLN4 (genomic instability driver gene) in the GU-like group was significantly higher compared with the level in the GS-like group (Fig. 2D, *P* < 0.001).

Functional enrichment analysis was performed to predict their potential functions and explore whether the potential biological functions and signaling pathways related with 189 lncRNAs are related to genomic instability. A lncRNA-mRNA co-expression network, with nodes representing lncRNA and mRNA, and lines representing co-expression relationship between them was constructed (Fig. 2E). GO enrichment analysis of lncRNA co-expressed genes showed that the genes in the network were significantly enriched in signal release, regulation of peptide secretion and hormone transport (Fig. 2F). KEGG pathway analysis showed that the genes were significantly enriched in MAPK signaling pathway, RAS signaling pathway and cAMP signaling pathway mostly related to genomic instability (Fig. 2G). These findings indicate that the 189 lncRNAs are associated with genomic instability.

### Construction of prognostic models for genomic instability lncRNAs and evaluation of their predictive performance

To further explore the prognostic role of these genomic instability-related lncRNAs, 171 pancreatic adenocarcinoma patients from the TCGA database were randomly divided into a training set (87 patients) and testing set (84 patients). Analysis was performed using Chi-square test to verify that there was no clinical bias (Table 1, *P* > 0.05). The relationship between 189 lncRNAs associated with genomic instability and overall survival of the training group was explored by univariate Cox regression. The findings showed that 5 lncRNAs were significantly correlated with prognosis of pancreatic adenocarcinoma patients (Figure S1). A prognostic model of lncRNAs associated with genomic instability was constructed based on the coefficient of multivariate Cox analysis and expression level of prognostic lncRNAs (GILncSig score = (0.1969 × expression level of LINC02577) + (0.0138 × expression level of LINC01133) + (-0.7284 × expression level of AC107464.2). Positive coefficients of LINC02577 and LINC01133 in GILncSig indicated that their overexpression may be a risk factor for pancreatic adenocarcinoma, whereas the negative coefficients of AC107464.2 implied that the lncRNA may be a protective factor for pancreatic adenocarcinoma.

**Table 1:** Statistical analysis of clinical preference of sample grouping

Covariates	Type	Total	Train	Test	*P* value
age	< = 65	90(52.63%)	48(55.17%)	42(50%)	0.600
	> 65	81(47.37%)	39(44.83%)	42(50%)	
gender	FEMALE	78(45.61%)	43(49.43%)	35(41.67%)	0.387
	MALE	93(54.39%)	44(50.57%)	49(58.33%)	
grade	G1-2	120(70.18%)	61(70.11%)	59(70.24%)	1.000
	G3-4	49(28.65%)	25(28.74%)	24(28.57%)	
	unknow	2(1.17%)	1(1.15%)	1(1.19%)	
stage	Stage I-II	161(94.15%)	80(91.95%)	81(96.43%)	1.000
	Stage III-IV	7(4.09%)	4(4.6%)	3(3.57%)	
	unknow	3(1.75%)	3(3.45%)	0(0%)	
T	T1-2	28(16.37%)	12(13.79%)	16(19.05%)	0.513
	T3-4	141(82.46%)	73(83.91%)	68(80.95%)	
	unknow	2(1.17%)	2(2.3%)	0(0%)	
M	M0	77(45.03%)	41(47.13%)	36(42.86%)	0.736
	M1	4(2.34%)	3(3.45%)	1(1.19%)	
	unknow	90(52.63%)	43(49.43%)	47(55.95%)	
N	N0	47(27.49%)	21(24.14%)	26(30.95%)	0.326
	N1	119(69.59%)	65(74.71%)	54(64.29%)	
	unknow	5(2.92%)	1(1.15%)	4(4.76%)	

**Fig. 3:**
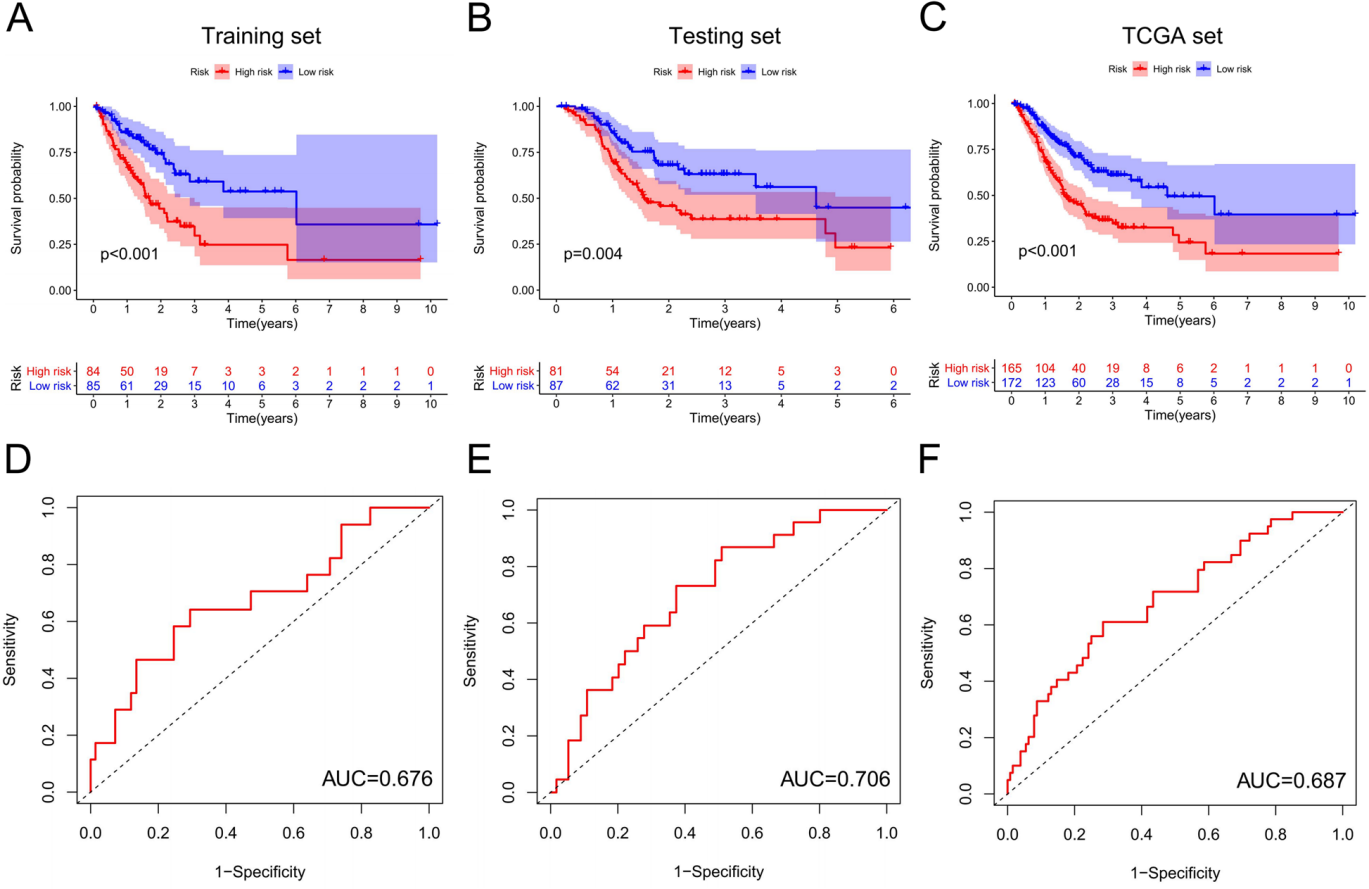
Evaluation and validation predictive performance of GInLncSig on OS in PAAD patients. Kaplan–Meier survival curves for high-risk and low-risk subsets were plotted according to GInLncSig scores in the training set **A**, test set **C**, and TCGA set **E**. Patients in the low-risk group showed longer survival compared with patients in the high-risk group (log-rank test, *P* < 0.05). The ROC curve of 1-year survival prediction of GInLncSig in training set **B**, test set **D** and TCGA set **F**

Patients were divided into high-risk and low-risk groups based on the risk value of the training set predicted by GILncSig. Kaplan–Meier analysis showed that the overall survival of patients in the high-risk group of the training set was significantly shorter compared with overall survival of patients in the low-risk group (Fig. 3A, *P* < 0.05). Survival prediction performance of GILncSig was verified using the testing set and TCGA set. The findings showed that the overall survival of low-risk patients was significantly better compared with the overall survival of high-risk patients (Fig. 3B-C, *P* < 0.05). The AUC value of the one-year survival prediction ROC curve of GILncSig for the training set was 0.067 (Fig. 3D), whereas the AUC values of the ROC curve for the testing set and TCGA set were 0.706 and 0.687, respectively (Fig. 3E-F). These findings indicate that the GInLncSig had a good survival prediction performance.

### Correlation analysis of GInLncSig and somatic mutation

**Fig. 4:**
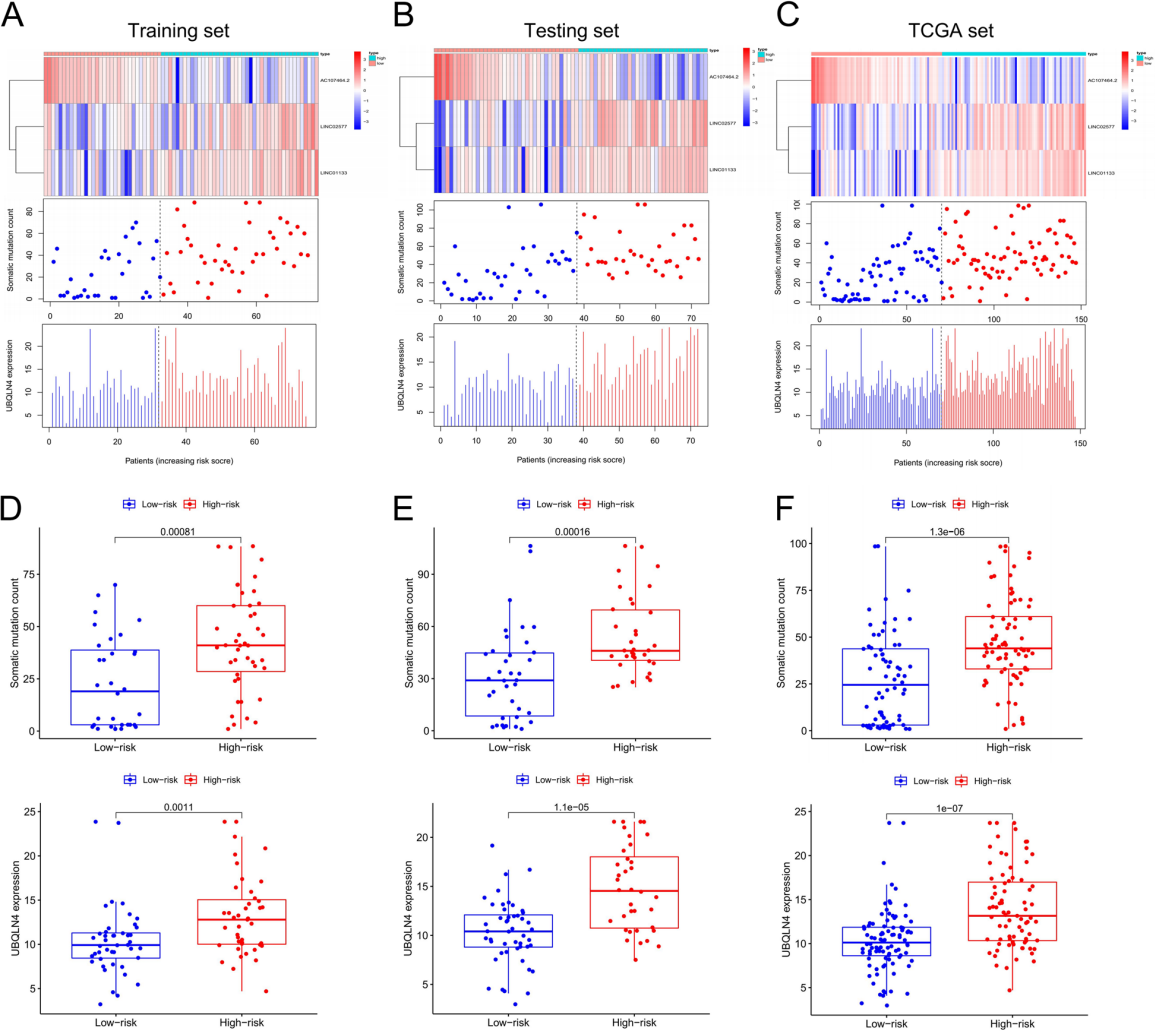
Relationship between GInLncSig and somatic mutation patterns in PAAD patients. Risk diagrams of training sets **A**, test sets **C**, and TCGA sets **E**, including lncRNA expression heat map, mutation distribution pattern, and UBQLN4 expression pattern. The expression levels of lncRNAs and UBQLN4 as well as somatic mutation levels changed with the increase in GInLncSig score. Somatic mutation counts and UBQLN4 expression level in the training set **B**, test set **D** and TCGA set **F** were significantly higher in the high-risk group than in the low-risk group

Analyses were performed for the training set, including generation of a heat map of lncRNAs expression, analysis of distribution of mutations in patients, and determination of expression level of UBQLN4 to explore whether GInLncSig was associated with somatic mutations. The findings showed that the expression level of lncRNAs, somatic mutation count of patients, and expression level of UBQLN4 were correlated with an increase in GInLncSig score (Fig. 4A). Expression levels of risk LINC01133 and LINC02577 were correlated with an increase in GInLncSig score. The expression level of the protective AC107464.2 decreased with an increase in GInLncSig score. On the contrary, somatic mutation count and expression level of UBQLN4 increased with increased GInLncSig score. Analysis using the testing set and TCGA set showed similar findings (Fig. 4B-C). Comparative analysis showed significant differences in somatic mutations and UBQLN4 expression levels between the high-risk and low-risk groups. The number of somatic mutations and expression level of UBQLN4 in the high-risk group were significantly higher than those in the low-risk group in the training set (Fig. 4D, *P* < 0.05). Analysis using the testing set and TCGA set showed similar findings (Fig. 4E-F, *p* < 0.05).

### Independent prognostic analysis and clinical grouping model validation of GInLncSig

Univariate and multivariate Cox regression analyses were performed on the three datasets (training, testing, and TCGA) to explore whether GInLncSig is an independent prognostic factor based on clinicopathological features. Findings from univariate and multivariate Cox regression analysis showed that GInLncSig was significantly correlated with overall survival of patients in the three datasets (Table 2, *P* < 0.05). Moreover, findings from multivariate Cox regression analysis showed that age was significantly correlated with overall survival in the training set and TCGA set, and stage was significantly correlated with overall survival in the testing set. Therefore, stratified analysis was performed to determine whether prognostic value of GInLncSig is independent of age and stage. Clinical stratification analysis of the prognosis of GInLncSig in the TCGA set showed that in the age <  = 65, male, G1 − 2 and stage I − II subgroups, the prognosis of patients in the low-risk group was better compared with that for the high-risk group (Fig. S2A-D). These findings indicate that GInLncSig is an independent prognostic factor associated with overall survival in pancreatic adenocarcinoma patients.

**Table 2:** Univariate and multivariate cox regression analysis of the GILncSig and overall survival in different patient sets

Variables	Univariate analysis				Multivariate analysis			
	HR	HR.95L	HR.95H	pvalue	HR	HR.95L	HR.95H	*p*value
Training set(*n* = 87)								
age	1.030	1.000	1.060	0.046	1.031	1.001	1.061	0.039
gender	0.793	0.442	1.423	0.437				
grade	1.467	0.992	2.170	0.055				
stage	0.671	0.317	1.423	0.299				
riskScore	1.645	1.310	2.066	< 0.001	1.677	1.320	2.131	< 0.001
Testing set (*n* = 228)								
age	1.021	0.990	1.053	0.192				
gender	0.992	0.542	1.815	0.980				
grade	1.373	0.879	2.145	0.163				
stage	2.063	1.221	3.485	0.007	1.781	1.017	3.118	0.044
riskScore	1.674	1.204	2.327	0.002	1.554	1.094	2.209	0.014
TCGA set (*n* = 171)								
age	1.027	1.006	1.049	0.012	1.027	1.005	1.050	0.016
gender	0.874	0.577	1.323	0.523				
grade	1.392	1.041	1.862	0.026	1.218	0.897	1.656	0.207
stage	1.365	0.936	1.991	0.106				
riskScore	1.619	1.368	1.917	< 0.001	1.615	1.360	1.919	< 0.001

### GInLncSig is compared with KRAS mutation status and lncRNA signatures

**Fig. 5:**
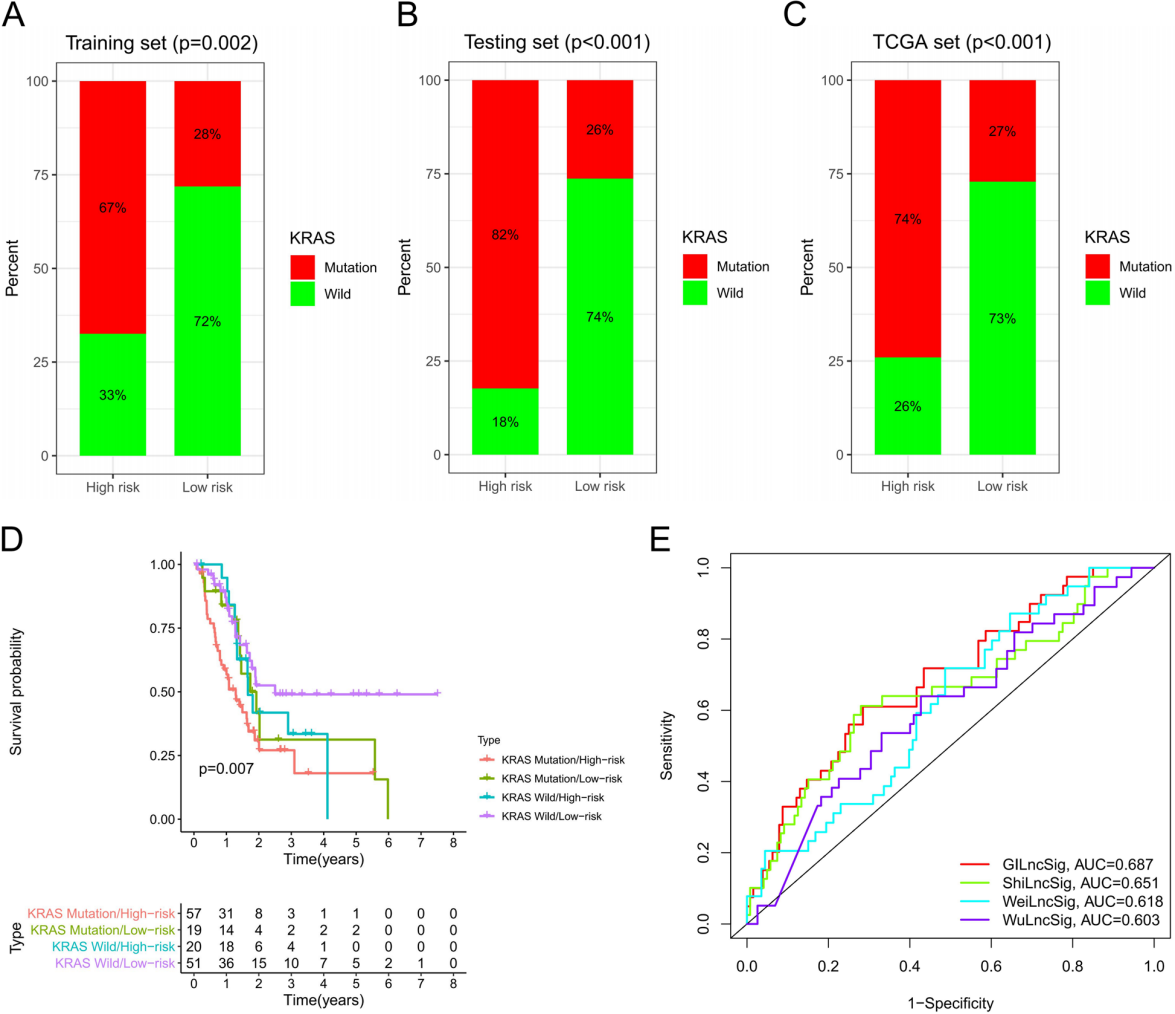
Prediction results of GInLncSig were superior to those of KRAS mutation status and existing lncRNA signatures. **A-C** The proportion of KRAS mutations in the high-risk group in the training set, test set, and TCGA set was significantly higher than that in the low-risk group (Chi-squared test, *P* < 0.05). **D** Co-survival analysis of GInLncSig and KRAS mutation status showed significant differences among the four groups (log-rank test, *P* < 0.001). **E** ROC curves for 1-year survival prediction of GInLncSig was better compared with the performance of the 3 previously reported lncRNA signatures

Further analysis was conducted to explore the relationship between GInLncSig and KRAS mutation status. Differences between the high-risk and low-risk groups in the three datasets were determined using Chi-square test. The findings showed that the proportion of KRAS mutation in the high-risk group was significantly higher than that in the low-risk group for the three datasets (Fig. 5A-C, *P* < 0.05).

These findings indicate that GInLncSig is correlated with KRAS mutation status and is a potential mutation marker of KRAS gene. Co-survival analysis of GInLncSig and KRAS mutation status was performed as follows, KRAS mutation /High − risk, KRAS mutation /Low − risk, KRAS wild /High − risk, and KRAS wild /Low − risk. The findings showed significant differences among the four groups (Fig. 5D, *P* < 0.05). In addition, significant differences in survival between high-risk and low-risk KRAS wild-type patients were observed. These findings imply that GInLncSig has greater prognostic significance compared with the KRAS mutation status alone and can be used for identification of the intermediate subtype group in patients with KRAS wild-type.

Further, TCGA pancreatic adenocarcinoma patient cohort was used to compare the prediction performance of GILncSig with three recently published lncRNA signatures, including 3-lncRNA reported by Shi et al. (ShilncSig), 9-lncRNA reported by Wei et al. (WeilncSig) and 3-lncRNA reported by Wu et al. (WulncSig). The findings showed that the AUC of GILncSig at 1 year of OS was 0.687, which was higher compared with the AUC for ShiLncSig (AUC = 0.651), WeiLncSig (AUC = 0.618) and WuLncSig (AUC = 0.603) (Fig. 5E). These findings indicate that the prognostic performance of GILncSig in predicting survival was better than the performance of the 3 previously reported lncRNA signatures.

### The immune landscape of high-/low-risk score groups

**Fig. 6:**
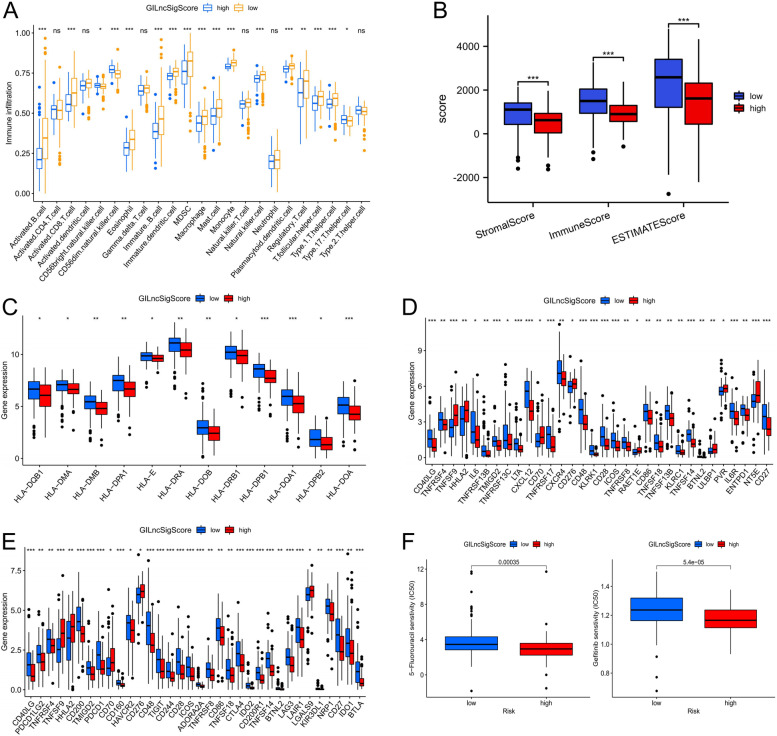
Immune landscape of high-/low- GILncSig score groups in the TCGA cohort. **A** The landscape of immune cell infiltration between two GILncSig score groups. **B** Stromal score, immune score and estimate score between two GILncSig score groups. **C-E** Gene expression of histocompatibility complexes, immunostimulators and immune checkpoints between two GILncSig score groups. **F** The chemotherapy response of two GILncSig score groups for two chemotherapy drugs. Statistical significance at the level of ns ≥ 0.05, ^∗^ < 0.05, ^∗∗^ < 0.01 and ^∗∗∗^ < 0.001

With the ssGSEA algorithm, we analyzed TME (tumor immune microenvironment) of high-/low-risk score groups in the TCGA cohort. (Fig. 6A). Most immune cells were highly infiltrated in the low-risk group, such as B cell, CD4 T and CD8 T, whereas CD56 bright and dim natural killer cells as well as Type17 T helper cells were highly infiltrated in the high-risk score group. TCGA expression profiles were used to calculate stromal scores, immune scores, and ESTIMATE scores for tumor tissues. As shown in Fig. 6B, compared with the high-risk score group, the samples of the low-risk score group also showed significantly higher estimated scores, stromal scores and immune scores. The expression levels of major histocompatibility complexes and immunostimulators tended to be higher in low-risk score group (Fig. 6C-D).

Subsequently, we examined whether the high-/low-risk score groups had a significant correlation with the immunotherapy effect. Compared with the high-risk score group, low-risk score group expression levels of major immune checkpoint molecules were significantly higher. (Fig. 6E). The IC50 values of 5-FU and Gefitinib were higher in low-risk score group than in high-risk score group (Fig. 6F). In summary, these results indicated that low-risk patients responded better to immunotherapy, but had little effect on chemotherapeutic drugs.

**Fig. 7:**
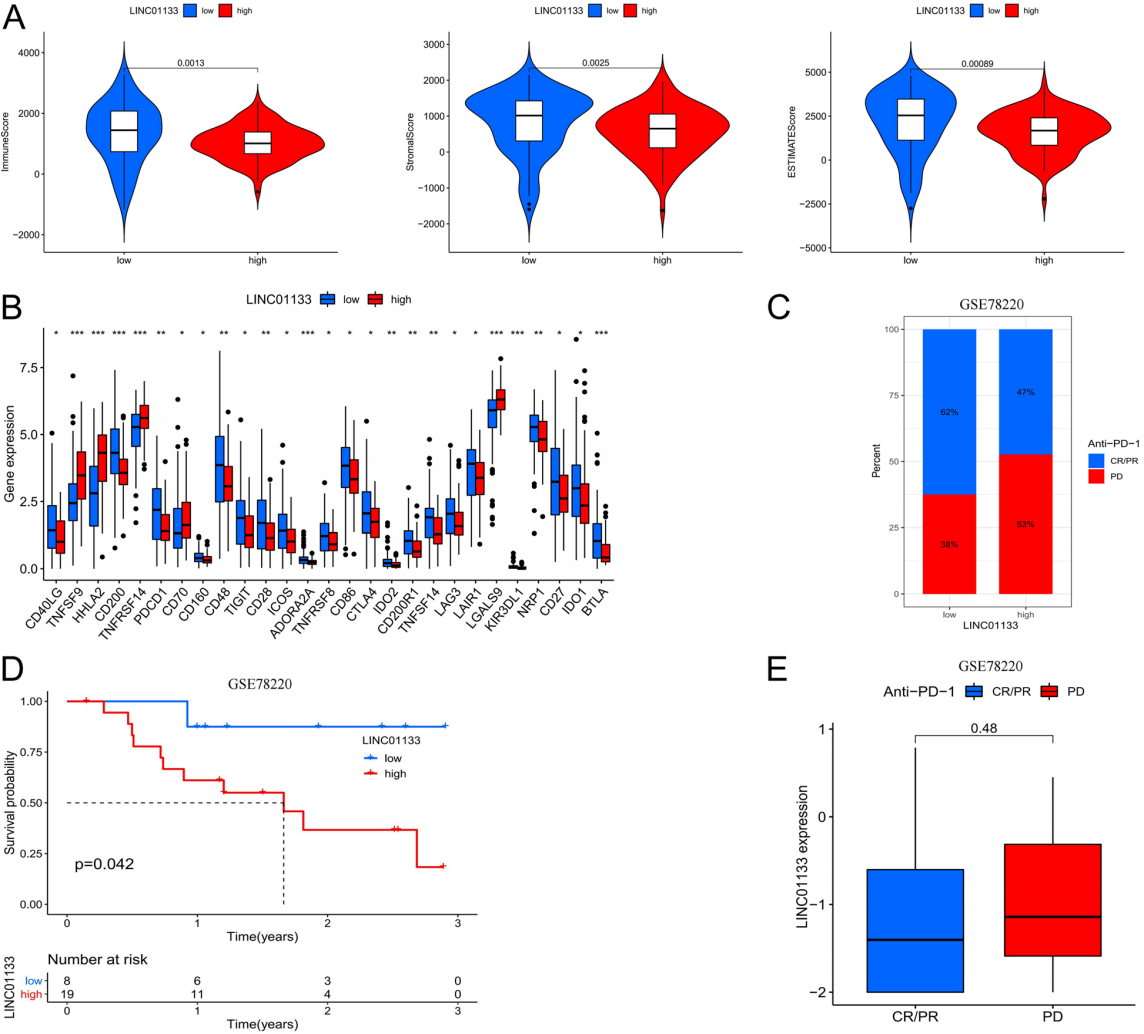
Immunotherapeutic benefits of LINC01133. **A** Immune score, stromal score and estimate score between high-/low- LINC01133 groups. **B** Gene expression of immune checkpoints between high-/low- LINC01133 groups. **C** Bar graph showed the distribution of PD and CR/PR in high- / low- LINC01133 groups. **D** Kaplan–Meier curve of OS for patients with high and low LINC01133 groups for GSE78220 cohort. **E **Boxplot graph illustrated the expression level of LINC01133 between PD and CR/PR groups. Statistical significance at the level of ns ≥ 0.05, ^∗^ < 0.05, ^∗∗^ < 0.01 and ^∗∗∗^ < 0.001. PD: progressive disease; CR: complete response; PR: partial response

However, we analyzed multiple immunotherapy datasets and found that LINC01133 expression data were found only in the GSE78220 dataset of anti-PD-1 therapy. Therefore, we analyzed the relationship between LINC01133 and anti-PD-1 therapy. We compared LINC01133 expression with immunological scores and immune checkpoints, and obtained results similar to the GILncSig score. As shown in Fig. 7A, compared with the high LINC01133 expression group, the samples of the low LINC01133 expression group also showed significantly higher estimated scores, stromal scores and immune scores. The expression levels of major immune checkpoint molecules in low LINC01133 expression group were distributed at the significantly higher(Fig. 7B).

The high LINC01133 expression patients possess a higher percentage of PD (progressive disease), and CR/PR (complete response/partial response) more happened in patients with low LINC01133 expression (Fig. 7C). In the GSE78220, low-LINC01133 patients showed longer OS than high-LINC01133 patients(Fig. 7D). However, the expression of LINC01133 did not differ significantly between the PD and CR/PR groups (Fig. 7E). These results implied that the low LINC01133 expression group responded better to immunotherapy.

### The 3 lncRNAs are correlated with clinical characteristics of pancreatic adenocarcinoma patients

Differences in expression levels of the three lncRNAs (LINC02577, LINC01133 and AC107464.2) were explored in tumor tissues and normal tissues. The findings showed that expression levels of LINC02577, LINC01133 and AC107464.2 in pancreatic adenocarcinoma tissues were significantly higher than in normal tissues (Fig. S3A, *P* < 0.05). Upregulation of LINC02577 and LINC01133 was significantly correlated with T stage, DSS event, OS event and histologic grade. Further, downregulation of AC107464.2 expression was significantly correlated with T stage, DSS event and OS event (Fig. S3B-E, *p* < 0.05). These findings further indicate that high expression of LINC02577 and LINC01133 is a risk factor in pancreatic adenocarcinoma patients, and high expression of AC107464.2 plays a protective role. Prediction of Normal and Tumor outcomes showed that LINC02577 had the highest accuracy (AUC = 0.967), whereas LINC01133 showed lower accuracy (AUC = 0.962) compared with LINC02577. The prediction ability of AC107464.2 was the lowest (AUC = 0.577) (Fig. S3F, *P* < 0.05).

In addition, according to Moffitt and Colisson's method, TCGA-PAAD samples were divided into two subtypes and three subtypes, respectively (Fig. S4A-B, D-E). Three lncRNAs (LINC02577, LINC01133 and AC107464.2) were differentially expressed in Moffitt and Collisson's classification (Fig. S4C,F). At same time, It was found that the expression of LINC02577 and LINC01133 increased in the blood extracellular vesicle of tumor patients, and LINC02577 was statistically significant (Fig. S4G). However, the expression of AC107464.2 was not detected. As shown in Fig. S4H, the expression levels of LINC01133 and LINC02577 in KRAS-Mut and KRAS + TP53-Mut samples were significantly higher than those in KRAS-Wild. The expression level of AC107464.2 in KRAS-Mut samples was significantly lower than that in KRAS-Wild samples.

### GSEA analysis of the 3 lncRNAs

**Fig. 8:**
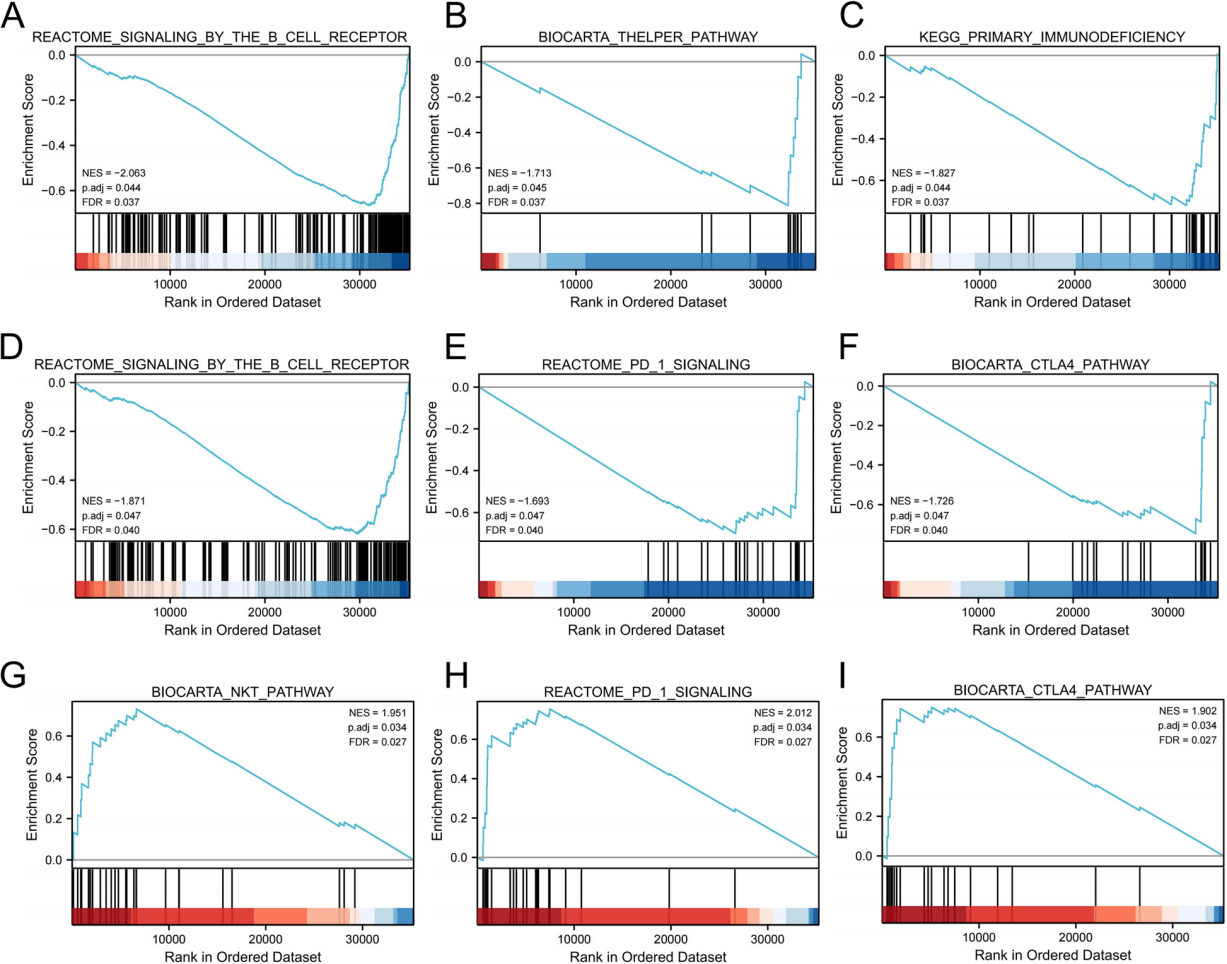
Gene set enrichment analysis of 3 lncRNAs. GSEA results showing signaling by the b cell receptor bcr **A**, t-helper pathway **B** and primary immunodeficiency **C** are differentially enriched in LINC02577-related PAAD. Signaling by the b cell receptor bcr **D**, PD-1 signaling **E** and CTLA4 pathway **F** are differentially enriched in LINC01133-related PAAD. PD-1 signaling **G**, CTLA4 pathway **H** and NKT pathway **I** are differentially enriched in AC107464.2-related PAAD

In explore the molecular mechanism of the 3 lncRNAs in the development and progression of pancreatic adenocarcinoma, GSEA analysis was performed to identify signaling pathways associated with the 3 lncRNA in the high-expression and low-expression groups of pancreatic adenocarcinoma. The findings showed that high expression of LINC02577 inhibited signaling by the B cell receptor BCR, t-helper pathway and primary immunodeficiency. Overexpression of LINC01133 inhibited signaling by the B cell receptor BCR, PD-1 signaling, and CTLA4 pathways. However, high expression of AC107464.2 activated PD-1 signaling, CTLA4 pathway, and NKT pathway (Fig. 8A-I and Table 3). These findings indicate that these lncRNAs are implicated in immunotherapy.

**Table 3:** Gene sets enriched in 3-lncRNA

LncRNA	Gene set name	NES	p.adjust	qvalues
LINC02577	REACTOME_SIGNALING_BY_THE_B_CELL_RECEPTOR_BCR	-2.063	0.044	0.037
	BIOCARTA_THELPER_PATHWAY	-1.713	0.045	0.037
	KEGG_PRIMARY_IMMUNODEFICIENCY	-1.827	0.044	0.037
LINC01133	REACTOME_SIGNALING_BY_THE_B_CELL_RECEPTOR_BCR	-1.871	0.047	0.040
	REACTOME_PD_1_SIGNALING	-1.693	0.047	0.040
	BIOCARTA_CTLA4_PATHWAY	-1.726	0.047	0.040
AC107464.2	BIOCARTA_NKT_PATHWAY	1.951	0.034	0.027
	REACTOME_PD_1_SIGNALING	2.012	0.034	0.027
	BIOCARTA_CTLA4_PATHWAY	1.902	0.034	0.027

### The 3 lncRNAs are correlated with immune cell infiltration

**Fig. 9:**
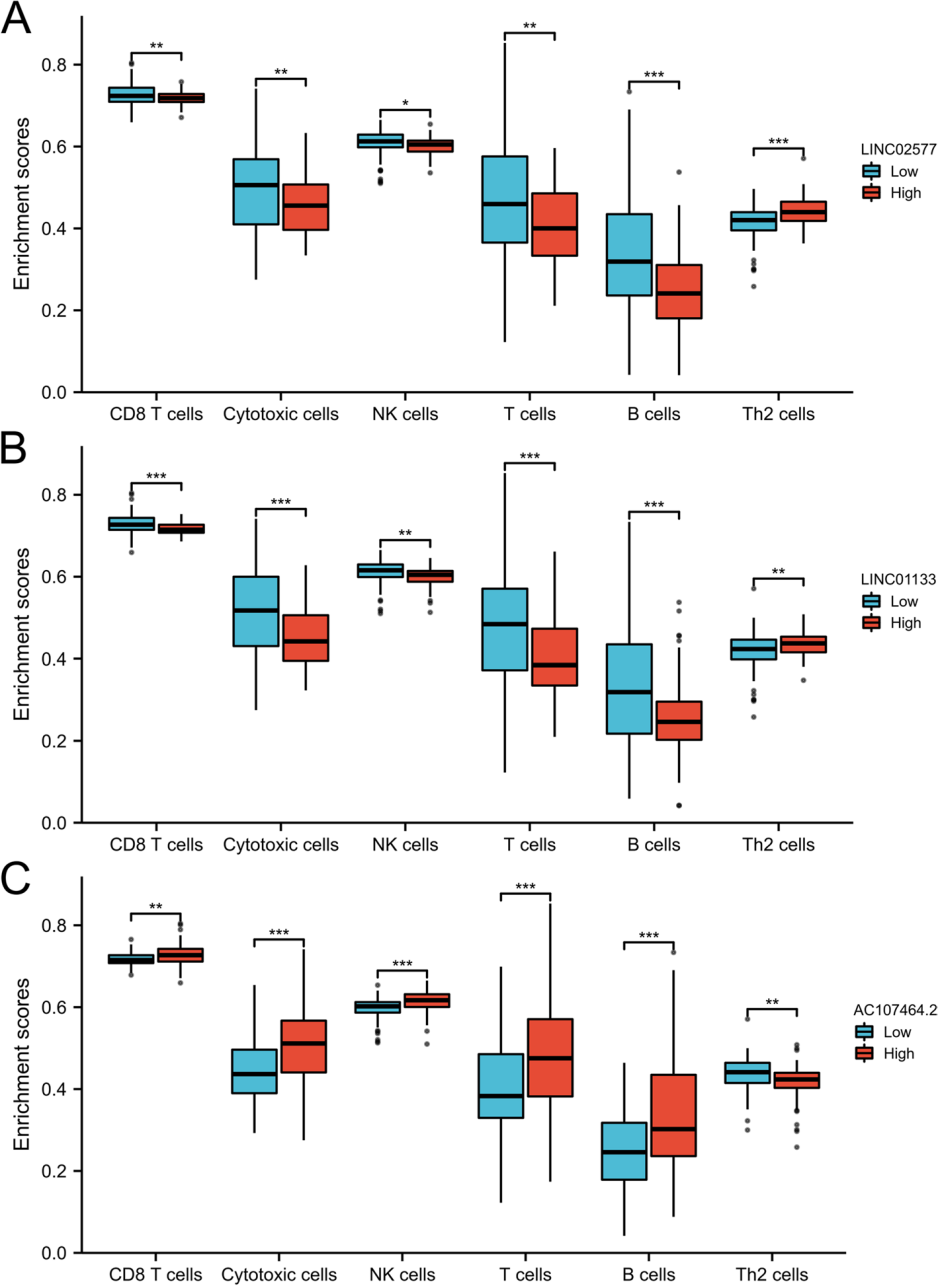
Correlation between the relative enrichment score of immune cells and expression level of the 3 lncRNAs in PAAD. **A-B** Infiltration levels of CD8 T cells, cytotoxic cells, NK cells, T cells and B cells in the high expression group of LINC02577 and LINC01133 were significantly lower compared with those in the low expression group; Infiltration level of Th2 cells in the high expression group of LINC02577 and LINC01133 was significantly higher compared with that in the low expression group. Level of immune cell infiltration in AC107464.2 high and low expression group showed an opposite trend **C**

The correlation between 3-lncRNA and 24 immune cells was performed based on the findings from GSEA enrichment analysis. The findings showed that LINC02577 was significantly positively correlated with infiltration level of Th2 cells and NK CD56 bright cells, and negatively correlated with infiltration levels of TFH, pDC and B cells (Fig. S5A and Table S2). Furthermore, LINC01133 was significantly positively correlated with infiltration levels of Th2 cells and macrophages, and negatively correlated with infiltration levels of TFH, pDC and Tgd (Fig. S5B and Table S3). Moreover, AC107464.2 was significantly positively correlated with infiltration level of TFH, pDC and B cells, and negatively correlated with infiltration level of Th2 cells (Fig. S5C and Table S4). Further analysis showed that infiltration levels of CD8 T cells, cytotoxic cells, NK cells, T cells and B cells may have protective effects on pancreatic adenocarcinoma patients. Infiltration levels of these cells were significantly lower in the high expression group of LINC02577 and LINC01133.compared with those in the low expression group. The infiltration level of Th2 cells which may be a risk factor for pancreatic adenocarcinoma patients was significantly higher in the high expression group of LINC02577 and LINC01133 compared with the low expression group (Fig. 9A-B, *P* < 0.05). Notably, the infiltration level of immune cells in the AC107464.2 high and low expression group showed an opposite trend (Fig. 9C, *P* < 0.05).

**Fig. 10:**
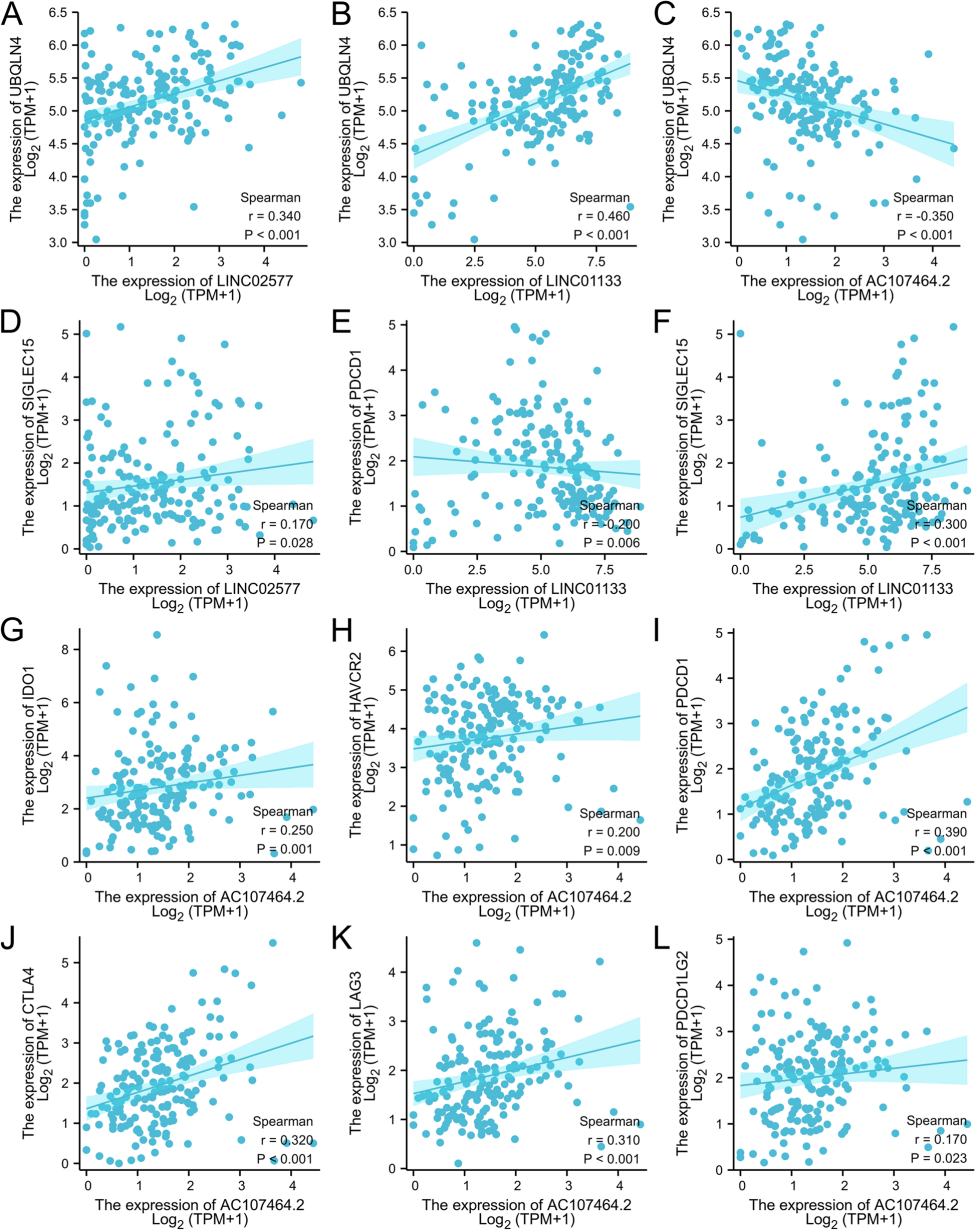
Spearman correlation of the 3 lncRNAs with UBQLN4 and immune checkpoints. LINC02577 and LINC01133 were positively correlated with expression level of UBQLN4, the driver gene of genomic instability **A-B**. AC107464.2 was negatively correlated with expression of UBQLN4 **C**. LINC02577 was significantly positively correlated with immune checkpoint SIGLEC15 **D**. LINC01133 was significantly negatively correlated with immune checkpoint PDCD1 **E** and positively correlated with immune checkpoint SIGLEC15 **F**. AC107464.2 was positively correlated with immune checkpoints IDO1, HAVCR2, PDCD1, CTLA4, LAG3, and PDCD1LG2 **G-L**

Further, correlation analysis of LINC02577, LINC01133 and AC107464.2 with genomic instability driver gene UBQLN4 and immune checkpoints were performed. The findings showed that LINC02577 and LINC01133 were significantly positively correlated with expression level of UBQLN4, whereas AC107464.2 was significantly negatively correlated with expression level of UBQLN4 (Fig. 10A-C, *P* < 0.05). In addition, a significant positive correlation was observed between LINC02577 and immune checkpoint SIGLEC15 (Fig. 10D). LINC01133 was significantly negatively correlated with immune checkpoint PDCD1 and positively correlated with immune checkpoint SIGLEC15 (Fig. 10E-F). AC107464.2 was positively correlated with immune checkpoints IDO1, HAVCR2, PDCD1, CTLA4, LAG3, and PDCD1LG2 (Fig. 10G-L).

**Fig. 11:**
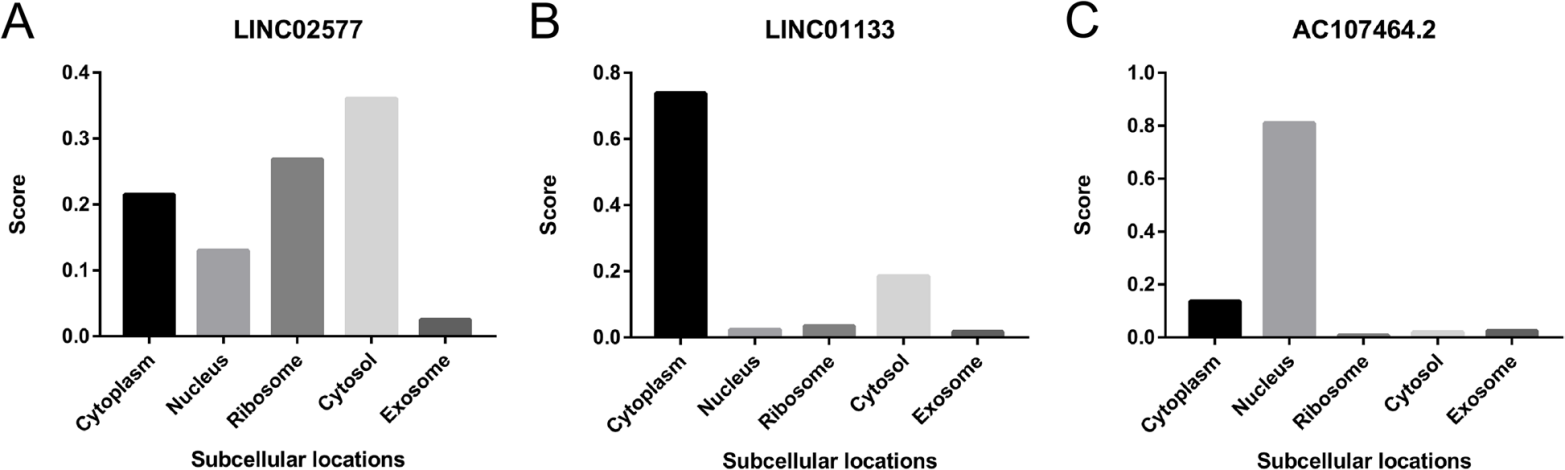
Subcellular localization of 3-lncRNA was predicted using LncLocator database. LINC02577 was mainly localized in cytosol and ribosome **A**. LINC01133 was mainly localized in cytoplasm **B**. AC107464.2 was mainly localized in nucleus **C**

The subcellular localization of lncRNAs was predicted using lncLocator database. The findings showed that LINC02577 was mainly localized in cytosol and ribosome, whereas LINC01133 was mainly localized in the cytoplasm. The findings showed that AC107464.2 was mainly localized in the nucleus (Fig. 11A-C). These findings indicate that LINC02577 and LINC01133 are localized in the cytoplasm and regulate genomic instability primarily at post-transcriptional levels, through ceRNA mechanism. AC107464.2 was localized in the nucleus and regulates genomic instability primarily at the transcriptional level, through mechanisms such as interaction with transcription factors.

## Discussion

Genome instability plays a significant role in modulating a variety of cancer-related characteristics in tumors [13]. Mutations promote cancer development, tumor progression, and resistance to treatment, thus genomic instability is a key diagnostic and prognostic marker in cancer [45, 46]. Advances in immunotherapy in cancer medicine have increased interest in understanding the mechanisms by which patients respond or are resistant to immunotherapy. Genomic changes associated with cancer development play key roles in immunotherapy response, and may result in genomic instability [47]. Furthermore, correlated between genomic instability with neoantigen production is the basis of treatment of cancer with immune checkpoint blocking inhibitors [47].

### IncRNAs plays an important role in genomic instability

Previous studies report that abnormal transcriptional and epigenetic regulation affects genomic instability [48]. Several mRNA and miRNA signatures have been developed to determine the degree of genome instability in cancer [49, 50]. Previous studies report that lncRNAs are promising tumor biomarkers associated with genomic stability [51]. Pancreatic adenocarcinoma (PAAD) is characterized by the lowest survival rate in all cancer types [52]. Approximately 8% of PAADs are present with unresectable tumors at diagnosis, therefore, patients with PAAD mainly rely on systemic treatment [12]. However, the first-line PAAD chemotherapy and immunotherapy are not fully effective [53, 54]. Genomic instability has been reported in pancreatic adenocarcinoma, and identification of molecules associated with genomic instability in PAAD tissue can contribute to development of personalized and more effective pancreatic adenocarcinoma treatment strategies [11]. Previous studies report that genome-based instability can provide a basis for development of a promising strategy to restore anti-tumor immunity, thus providing novel therapeutic opportunities for cancer therapy [17]. Currently, only a few studies have explored the role of lncRNAs in PAAD genomic instability. The current study constructed a 3-lncRNA signature (GInLncRNAs) based on PAAD samples. Further, the study explored prognostic significance of the signature in PAAD patients and the relationship with tumor immunotherapy.

### Identification of genome instability associated lncRNAs and its clinical value

In this study, lncRNA expression profile was combined with the somatic mutation profile of pancreatic adenocarcinoma. A total of 189 lncRNAs associated with pancreatic adenocarcinoma genomic instability were identified. Functional enrichment analysis was performed based on co-expressed genes of the 189 lncRNAs. GO enrichment analysis showed that these co-expressed genes were significantly enriched in signal release, regulation of peptide secretion and hormone transport. Findings from KEGG pathway enrichment analysis showed that the co-expressed genes were significantly enriched in the MAPK signaling pathway, Ras signaling pathway and cAMP signaling pathway. De et al. [55] reported that MAPK signaling affects genomic instability by modulating p53 activity and controlling cell G2 checkpoints and DNA damage. A study by Graziano [56] reported that Ras induces genomic instability. Holcomb [57] reported cAMP-mediated regulation of melanoma genomic instability.

Further studies were conducted to explore whether the genomic instability-associated lncRNAs can predict clinical outcomes in patients with pancreatic adenocarcinoma. A lncRNA signature (GILncSig) comprising the three lncRNAs (LINC02577, LINC01133, and AC107464.2) associated with genomic instability was constructed. GILncSig grouped PAAD patients in the training set into two risk groups with significantly different survival. The findings were validated using the testing set and TCGA set. The findings showed that the GILncSig was significantly associated with somatic mutation and UBQLN4 expression, which are essential indicators of genomic instability. The number of somatic mutations and expression level of UBQLN4 in the high-risk group were significantly higher than those in the low-risk group. Notably, analysis using the testing set and TCGA set showed similar findings. A previous study reports that LINC01133 activates the Wnt signaling pathway to promote occurrence of pancreatic adenocarcinoma [58]. Wnt signaling pathway is highly correlated with somatic mutations [59]. Biological functions of LINC02577 and AC107464.2 have not been reported in previous studies. Functional analysis showed that LINC02577 and AC107464.2 modulate genomic instability related DNA damage and DNA repair, respectively. These findings indicate that the GILncSig constructed in the current study can predict prognosis in patients with pancreatic adenocarcinoma, and serve as an indicator for genomic instability in patients with pancreatic adenocarcinoma.

### Compared to KRAS mutation status alone, GinLncSig has greater prognostic significance

Analysis of the GILncSig score, showed that the KRAS mutation rate in the high-risk group was significantly higher compared with that of the low-risk group. This finding implies that GILncSig can be used to predict the KRAS mutation status. In addition, the findings of the current study showed that GILncSig accurately distinguished between different clinical outcomes in patients with KRAS wild-type. The survival time of KRAS wild-type patients in the low-risk group was significantly longer than that for KRAS mutant patients. On the contrary, the findings did not show significant difference between KRAS wild-type patients and KRAS mutant patients in the high-risk group. Significant difference in survival between high-risk and low-risk KRAS wild-type patients indicates that GILncSig has a higher prognostic significance compared with KRAS mutation status alone. Moreover, the GILncSig could identify intermediate subtype groups with partial KRAS function in KRAS wild-type patients. In addition, the findings showed that GILncSig had better prognostic performance in predicting survival compared with three previously reported lncRNA signatures.

### GILncSig may serve as a biomarker to predict immunotherapy response

It is well known that genomic instability is closely related to tumor immunity [60]. An investigation was conducted to determine the relationship between GILncSig scores and the infiltration of TME cells. Most immune cells were highly infiltrated in the low-risk score group, and the samples of the low-risk score group also showed significantly higher estimated scores, stromal scores and immune scores. In addition, the major histocompatibility complexes and immunostimulators are also up-regulated in low-risk score group. Thus, low-risk score group patients are associated with immune activation and have better PAAD outcomes; on the other hand, high-risk score group patients are associated with immunosuppression and have poor PAAD outcomes. In addition, compared with the high-risk score group, low-risk score group expression levels of major immune checkpoint molecules were significantly higher. Especially, malignant melanoma datasets with anti-PD-1 treatments demonstrated LINC01133 to be a reliable predictor. In summary, GILncSig may serve as a biomarker to predict immunotherapy response.

### Correlation between three lncRNAs and clinical characteristics

The three lncRNAs in GILncSig have not been studied yet, therefore, the biological functions and mechanisms of action of the three lncRNAs in pancreatic adenocarcinoma were predicted using bioinformatics tools. These findings provide a basis to further explore the roles of these lncRNAs. GTEX data was integrated for expression level analysis owing to the small number of adjacent normal tissues from TCGA. The findings showed that t expression levels of LINC01133 and AC107464.2 in pancreatic adenocarcinoma tissues were significantly higher compared with those in normal tissues. Studies report that high expression of LINC01133 in pancreatic adenocarcinoma plays a carcinogenic role [58], and LINC01133 can be transported to the extracellular space by pancreatic adenocarcinoma cells through exosomes to promote epithelial mesenchymal transformation of pancreatic duct adenocarcinoma [61]. The constructed model showed that LINC02577 and LINC01133 were risk factors, whereas AC107464.2 was a protective factor for pancreatic adenocarcinoma. LINC01133 is highly expressed in pancreatic adenocarcinoma, implying that AC107464.2 which plays a protective role occurs at a low expression level in pancreatic adenocarcinoma.

TCGA data of pancreatic adenocarcinoma was separately analyzed to avoid influence of combining with GETX data. The findings showed that expression of LINC02577 and LINC01133 was significantly higher in tumor samples compared with the level in the adjacent samples. Furthermore, the expression level of AC107464.2 was lower in tumor samples than in adjacent tissues. However, the difference in expression level of AC107464.2 between tumor and adjacent tissues was not statistically significant. In addition, expression levels of LINC02577 and LINC01133 were significantly correlated with T stage, DSS event, OS event and histologic grade, and the expression level of the two lncRNAs increased with an increase in tumor malignancy. The expression level of AC107464.2 was significantly correlated with T stage, DSS event and OS event, and the expression level decreased with an increase in tumor malignancy. These findings further confirm that high expression level of LINC02577 and LINC01133 in pancreatic adenocarcinoma patients is a risk factor, whereas high expression of AC107464.2 exhibits a protective role.

Early-stage pancreatic adenocarcinoma lacks specific clinical manifestations, therefore, diagnosis is challenging. Approximately 80% of pancreatic adenocarcinoma patients present with advanced stages at the time of diagnosis, thus treatment efficacy is reduced. Therefore, it is important to establish specific biomarkers for diagnosis of pancreatic adenocarcinoma. Analysis of the diagnostic ROC curve showed that LINC02577 and LINC01133 had high predictive power of 0.967 and 0.962, respectively in predicting Normal and Tumor outcomes. These findings indicate that LINC02577 and LINC01133 can be used as biomarkers for diagnosis of pancreatic adenocarcinoma, thus ensuring diagnosis of pancreatic adenocarcinoma.

### Three lncRNAs may affect the efficacy of immunotherapy

GSEA analysis showed that the 3 lncRNAs (LINC02577, LINC01133 and AC107464.2) were associated with immunotherapy-related signaling pathways. LINC01133 was implication in inhibition of PD-1 signaling and CTLA4 pathway. AC107464.2 was implicated in activation PD-1 signaling and CTLA4 pathway. Further analysis showed that the 3 lncRNA were associated with the level of tumor immune cell infiltration. In addition, the 3 lncRNA were significantly correlated with expression levels of UBQLN4, a genomic instability driver gene, and immune checkpoint-related genes. A significant correlation has been reported between genomic instability and immune checkpoint blocking [62]. These findings indicate that the 3 lncRNA may affect the efficacy of immunotherapy in patients with pancreatic adenocarcinoma by modulating genomic instability.

Subcellular localization prediction results showed that LINC02577 and LINC01133 were mainly localized in the cytoplasm, whereas AC107464.2 was localized in the nucleus. These findings indicate that LINC02577 and LINC01133 mainly regulate the expression of pancreatic adenocarcinoma-related genes at post-transcriptional level, to modulate genomic instability. On the contrary, AC107464.2 mainly regulates expression of related genes at transcriptional level, to modulate genomic instability.

The current study provides important insights on understanding genomic instability and prognosis in pancreatic adenocarcinoma patients, however, the study had a few limitations. Although the GILncSig was validated using the TCGA dataset, an independent dataset is required for validation of GILncSig to verify its accuracy. In addition, GILncSig was constructed using bioinformatics analysis. Therefore, further in vivo functional studies should be conducted to explore the regulatory mechanisms of GILncSig in modulating genomic instability and tumor immunity.

## Conclusion

This study proposes a mutant hypothesis-derived computational framework to identify genomic instability-related lncRNAs. This provides a key method and resource for further studies on the role of lncRNAs in genomic instability and immunotherapy. A genomic instability-related lncRNA signature was identified as an independent prognostic marker to stratify the risk subgroup of pancreatic adenocarcinoma patients by combining lncRNA expression profile with somatic mutation profile and clinical information of pancreatic adenocarcinoma. Prediction analyses indicated that GILncSig has important implications in genomic instability and making of treatment decisions in patients with pancreatic adenocarcinoma.

## Supplementary Information

**Additional file 1:**

## Data Availability

The data that support the findings of this work are obtainable from TCGA (https://portal.gdc.cancer.gov/) and other data in the paper can be obtained from the corresponding author based on reasonable request.

## Abbreviations

PAAD: Pancreatic adenocarcinoma
GInLncRNAs: Genome instability-related lncRNAs
GInLncSig: 3-LncRNA signature
GU: Genomic unstable
GS: Genomic stable
DSS: Disease specific survival
OS: Overall survival
GSEA: Gene set enrichment analysis
TME: Tumor immune microenvironment
