# Supplementary material for: Genome instability-related LINC02577, LINC01133 and AC107464.2 are lncRNA prognostic markers correlated with immune microenvironment in pancreatic adenocarcinoma
Source: BMC Cancer. 2023 May 12;23:430. doi: 10.1186/s12885-023-10831-4 (PMC10176692; doi:10.1186/s12885-023-10831-4)
Supplement: Supplementary file 1 — Additional file 1: [file 12885_2023_10831_MOESM1_ESM.docx]

**Supplementary material**

**
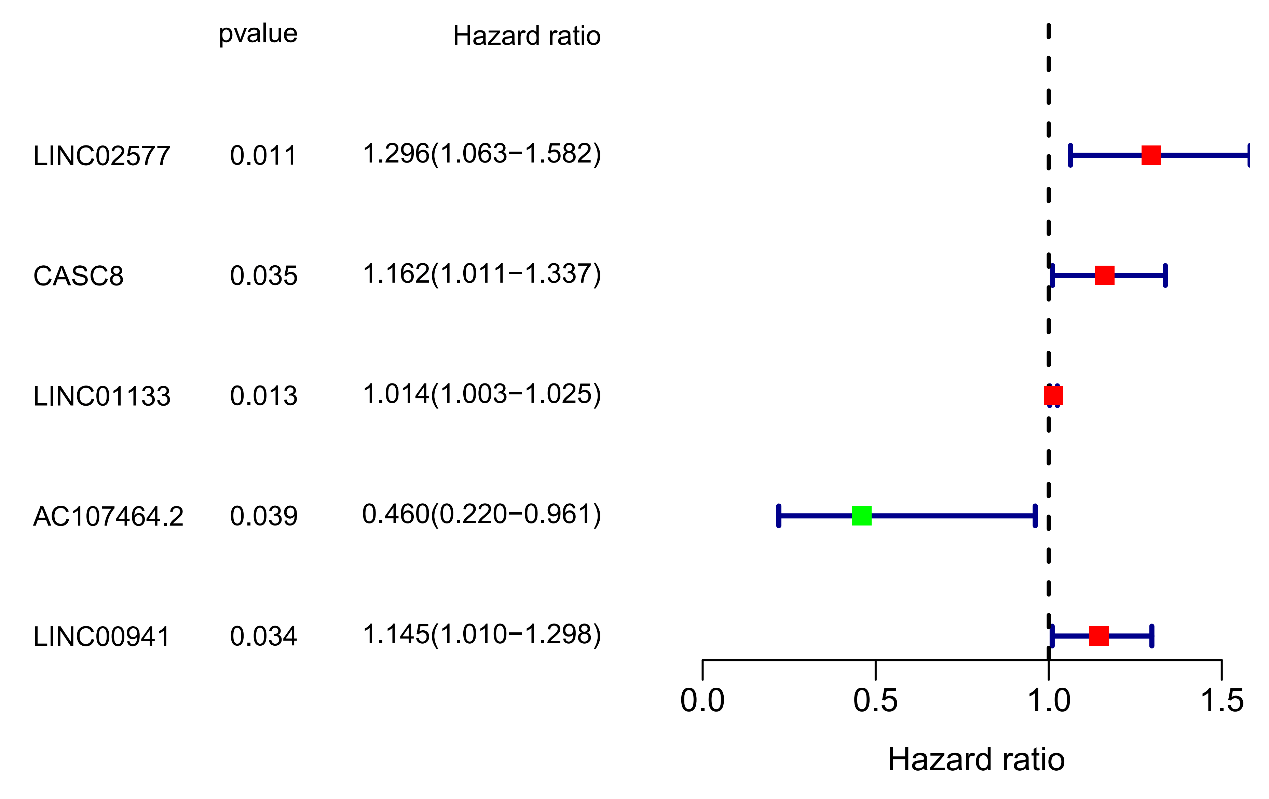
**

**Figure S1. Forest plot for five GInLncRNAs associated with OS of patients based on univariate Cox regression analyses**. One GInLncRNAs exhibited protecting role in survival of patients (AC107464.2 ), whereas the other four GInLncRNAs (LINC02577，CASC8，LINC01133 and LINC00941) were risk factors for survival of patients.

**
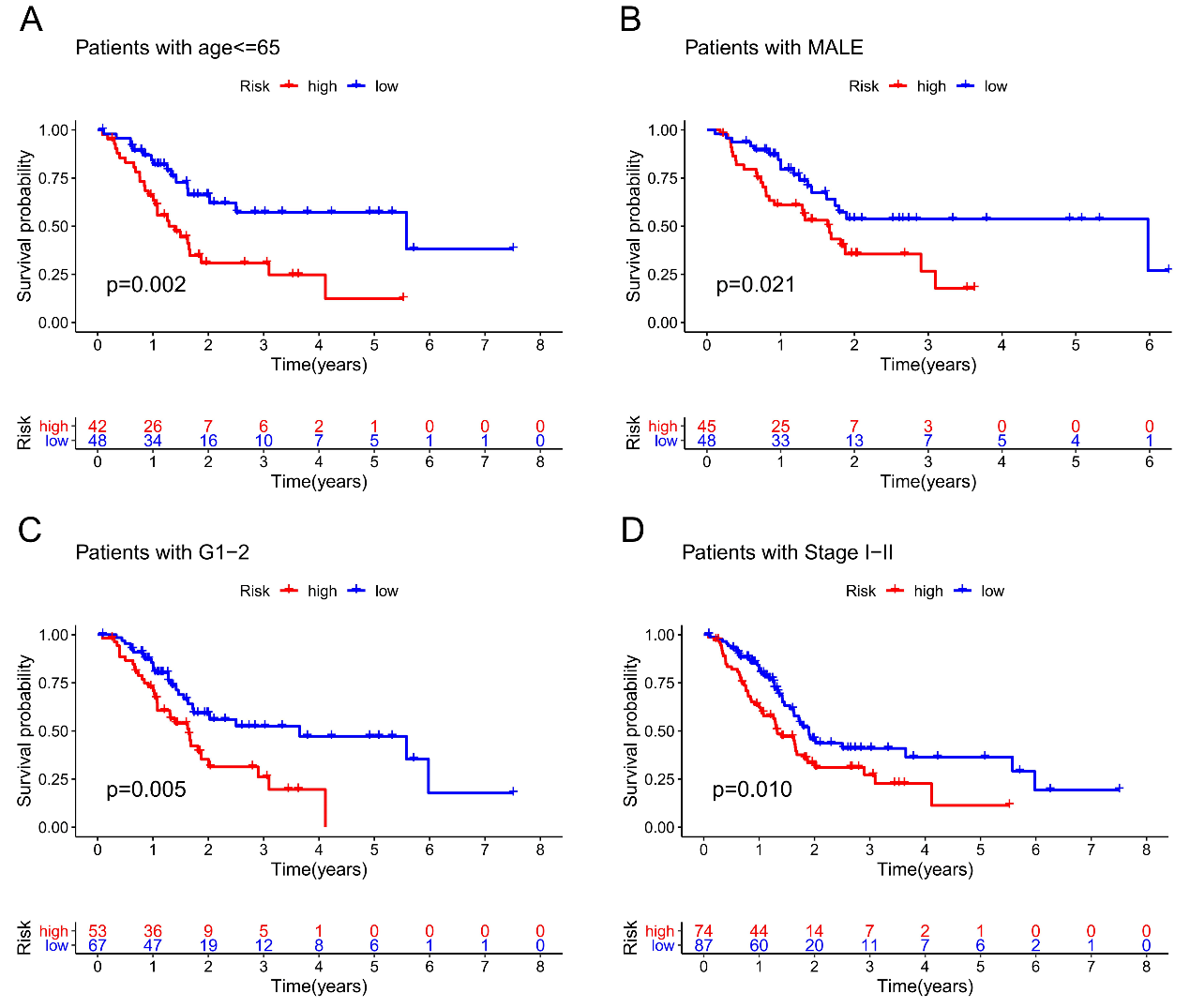
**

**Figure S2.** **Clinical stratification of PAAD patients in high-risk group and low-risk group.** Kaplan-Meier survival curve of patients under 65 years old (**A**), male (**B**), G1-2 tumor grade (**C**), I-II tumor stage (**D**) in high-risk and low-risk groups. In all clinical stratification subgroups, patients in the high-risk group had a shorter overall survival than those in the low-risk group(log-rank test, P < 0.05).


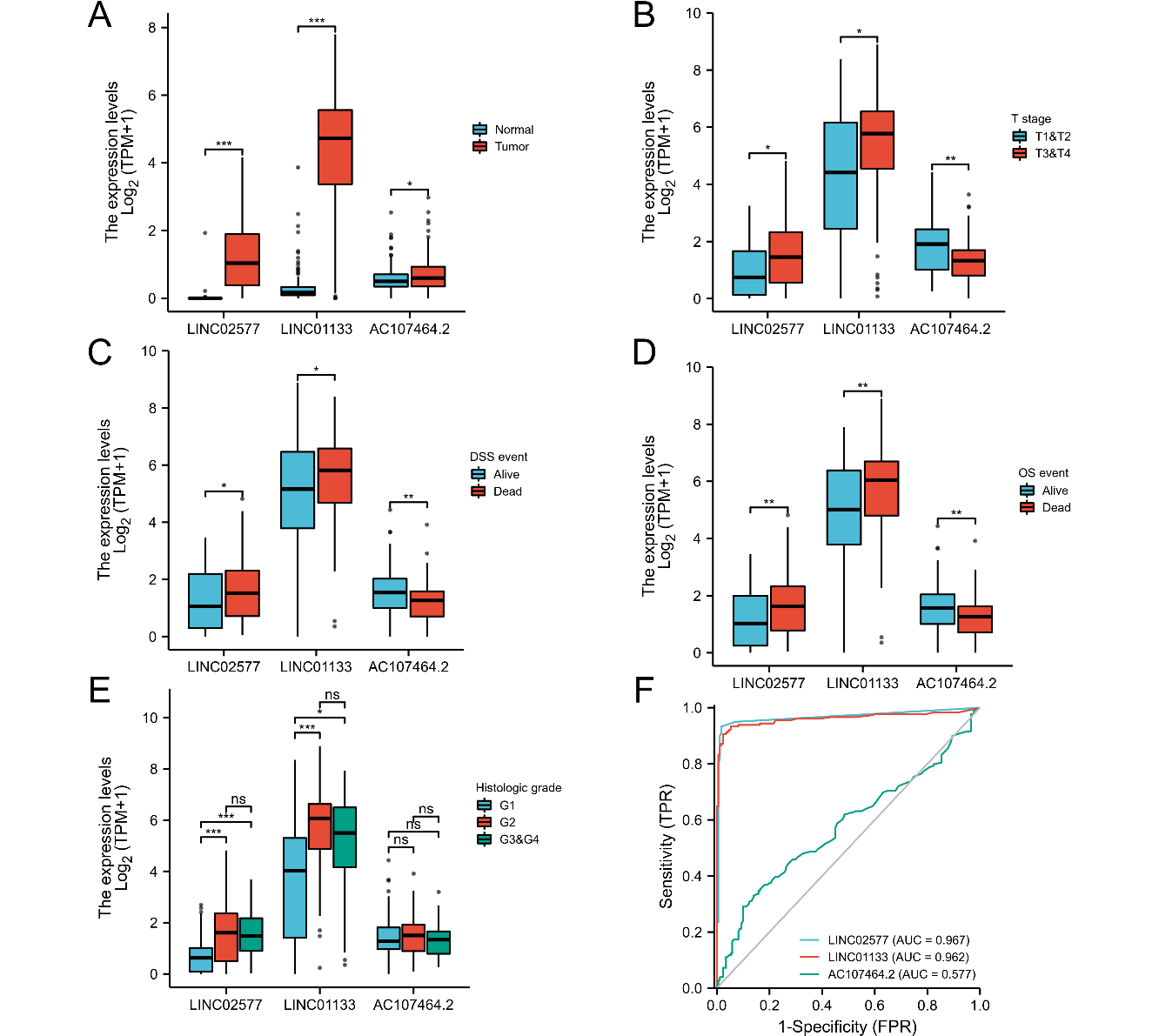


**Figure S3. Expression level of the 3 lncRNA, clinical correlation and diagnostic value analysis. (A**) LINC02577, LINC01133 and AC107464.2 showed significantly higher expression level in cancer tissues compared with levels in normal tissues. (**B-E**) LLINC02577, LINC01133 and AC107464.2 were significantly correlated with T stage, DSS event, OS event and histologic grade. (**F**) LINC02577 and LINC01133 had a high diagnostic value.


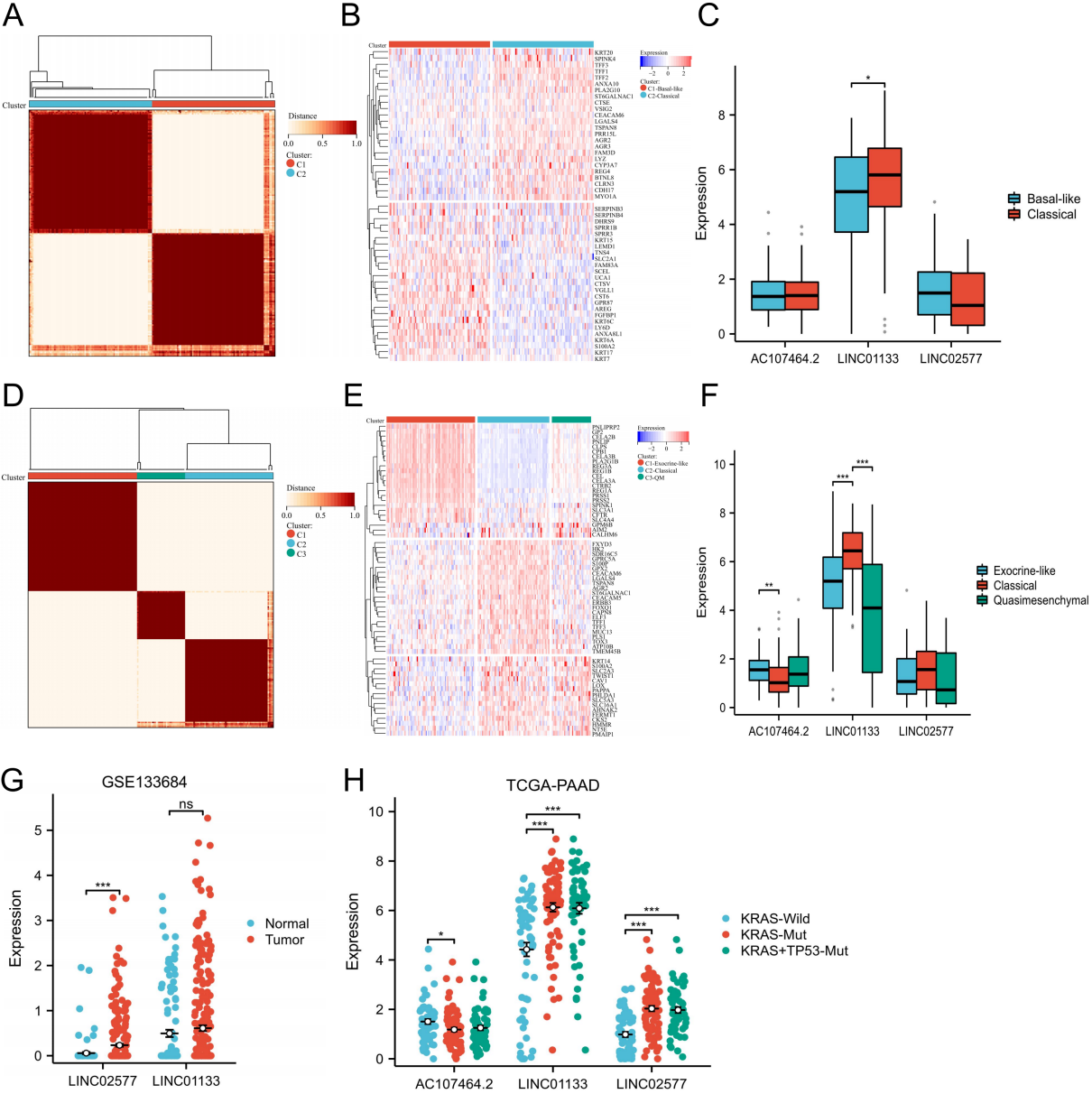


**Figure S4 The expression level of three lncRNAs.** (A-B) According to Moffitt's method, TCGA-PAAD samples were divided into two subtypes, namely classical (C2) and class base (C1). (C) The expression of LINC01133 in classical subtype was significantly higher than that in basal-like subtype. (D-E) According to Collisson's method, TCGA-PAAD samples were divided into three subtypes , namely classical (C2), quasimesenchymal (QM, C3) and exocrine-like (C1). (F) The expression of LINC01133 in classical subtype was significantly higher than that of quasimesenchymal (QM) and exocrine-like subtypes ; the expression of AC107464.2 in classical subtype was significantly lower than that in exocrine-like subtype. (G) The expression levels of three lncRNAs in blood samples. (H) The Expression level of lncRNAs in mutation and wild type samples. Wilcoxon rank sum test was selected for comparison between the two groups. Kruskal-Wallis test was selected for comparison among the three groups. ns: p>0.05, ^∗^: p<0.05, ^∗∗^: p<0.01 and ^∗∗∗^: p<0.001.


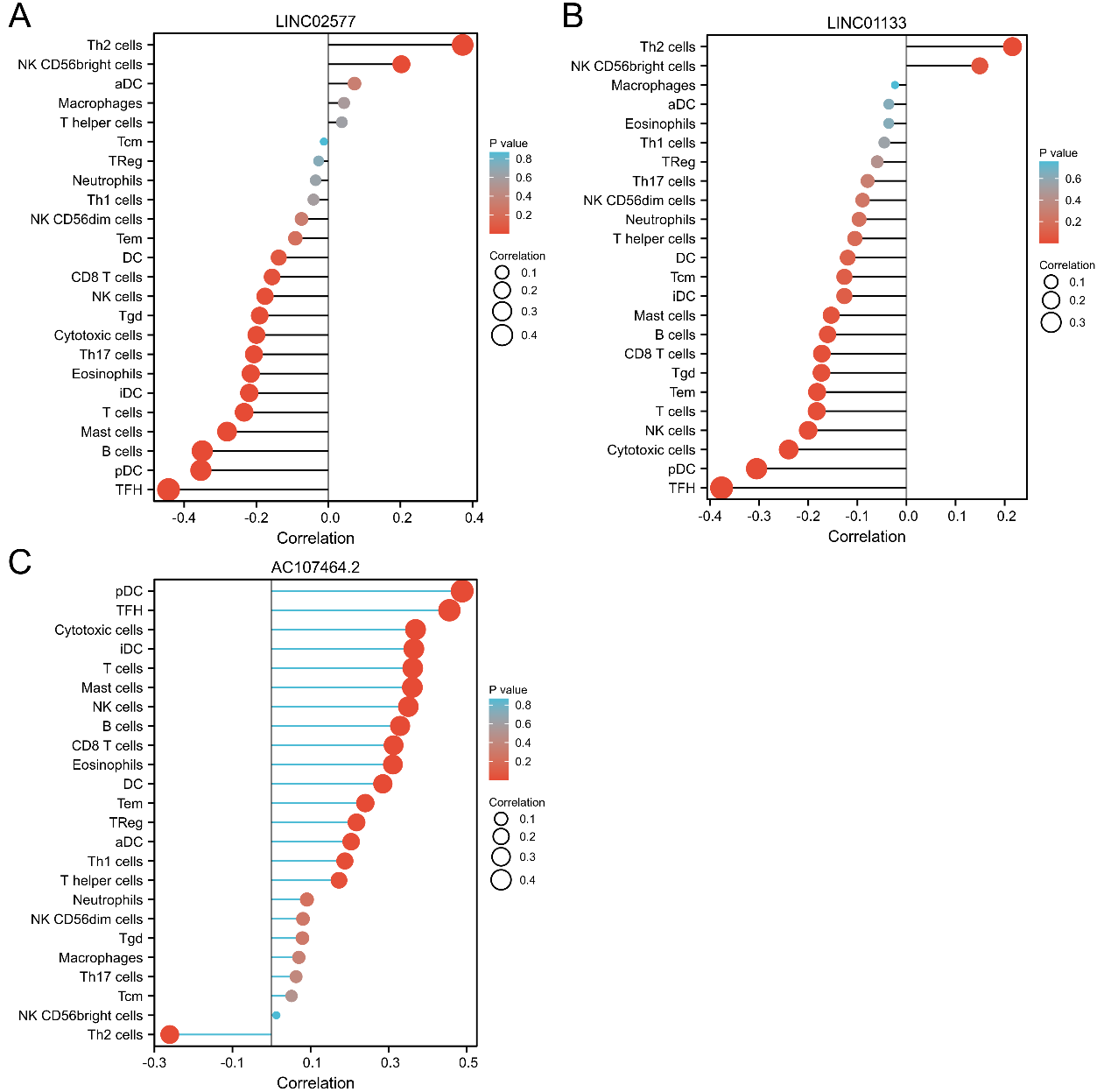


**Figure S5. Forest plot showing the correlation between expression of the 3 lncRNAs and immune cell subsets. (A)**LINC02577 was significantly positively correlated with Th2 cells and NK CD56 bright cells, and significantly negatively correlated with TFH, pDC and B cells. **(B)**LINC02577 showed a significant positive correlation with Th2 cells and macrophages, and a significant negative correlation with TFH, pDC and Tgd. (**C**)AC107464.2 was significantly positively correlated with TFH, pDC and B cells, and significantly negatively correlated with Th2 cells.

**Table S1.(xlsx) Identification of genome instability-related lncRNAs in PAAD patients.**

| **Table S2 The correlation between LINC01133 and tumor-infiltrating immune cells** | | |
| --- | --- | --- |
| Cells | Correlation | p value |
| Th2 cells | 0.307 | <0.001 |
| Macrophages | 0.22 | 0.003 |
| iDC | 0.109 | 0.146 |
| Th1 cells | 0.104 | 0.167 |
| DC | 0.06 | 0.424 |
| Eosinophils | 0.06 | 0.427 |
| aDC | 0.059 | 0.436 |
| Neutrophils | 0.043 | 0.571 |
| NK CD56bright cells | 0.039 | 0.609 |
| TReg | 0.029 | 0.705 |
| NK CD56dim cells | 0.01 | 0.89 |
| T helper cells | 0.002 | 0.976 |
| Th17 cells | -0.007 | 0.93 |
| CD8 T cells | -0.026 | 0.734 |
| Mast cells | -0.031 | 0.685 |
| T cells | -0.041 | 0.583 |
| NK cells | -0.044 | 0.561 |
| B cells | -0.059 | 0.435 |
| Tem | -0.066 | 0.379 |
| Tcm | -0.101 | 0.18 |
| Cytotoxic cells | -0.125 | 0.095 |
| Tgd | -0.183 | 0.014 |
| pDC | -0.233 | 0.002 |
| TFH | -0.294 | <0.001 |

| **Table S3. The correlation between AC107464.2 and tumor-infiltrating immune cells** | | |
| --- | --- | --- |
| Cells | Correlation | p value |
| TFH | 0.459 | <0.001 |
| pDC | 0.439 | <0.001 |
| B cells | 0.343 | <0.001 |
| T cells | 0.342 | <0.001 |
| Cytotoxic cells | 0.322 | <0.001 |
| Mast cells | 0.296 | <0.001 |
| CD8 T cells | 0.268 | <0.001 |
| Eosinophils | 0.255 | <0.001 |
| TReg | 0.246 | <0.001 |
| NK cells | 0.238 | 0.001 |
| iDC | 0.233 | 0.002 |
| DC | 0.211 | 0.005 |
| T helper cells | 0.193 | 0.01 |
| Tem | 0.192 | 0.01 |
| aDC | 0.172 | 0.022 |
| Th1 cells | 0.149 | 0.046 |
| Tgd | 0.108 | 0.15 |
| NK CD56bright cells | 0.096 | 0.203 |
| Tcm | 0.082 | 0.277 |
| NK CD56dim cells | 0.019 | 0.802 |
| Neutrophils | 0.014 | 0.851 |
| Th17 cells | 0.01 | 0.897 |
| Macrophages | 0.003 | 0.971 |
| Th2 cells | -0.31 | <0.001 |

| **Table S4. The correlation between AC107464.2 and tumor-infiltrating immune cells** | | |
| --- | --- | --- |
| Cells | Correlation | p value |
| TFH | 0.459 | <0.001 |
| pDC | 0.439 | <0.001 |
| B cells | 0.343 | <0.001 |
| T cells | 0.342 | <0.001 |
| Cytotoxic cells | 0.322 | <0.001 |
| Mast cells | 0.296 | <0.001 |
| CD8 T cells | 0.268 | <0.001 |
| Eosinophils | 0.255 | <0.001 |
| TReg | 0.246 | <0.001 |
| NK cells | 0.238 | 0.001 |
| iDC | 0.233 | 0.002 |
| DC | 0.211 | 0.005 |
| T helper cells | 0.193 | 0.01 |
| Tem | 0.192 | 0.01 |
| aDC | 0.172 | 0.022 |
| Th1 cells | 0.149 | 0.046 |
| Tgd | 0.108 | 0.15 |
| NK CD56bright cells | 0.096 | 0.203 |
| Tcm | 0.082 | 0.277 |
| NK CD56dim cells | 0.019 | 0.802 |
| Neutrophils | 0.014 | 0.851 |
| Th17 cells | 0.01 | 0.897 |
| Macrophages | 0.003 | 0.971 |
| Th2 cells | -0.31 | <0.001 |
